# Vegetable By-Products from Industrial Processing: From Waste to Functional Ingredient Through Fermentation

**DOI:** 10.3390/foods14152704

**Published:** 2025-07-31

**Authors:** Andrea Marcelli, Andrea Osimani, Lucia Aquilanti

**Affiliations:** Dipartimento di Scienze Agrarie, Alimentari e Ambientali (D3A), Università Politecnica delle Marche, Via Brecce Bianche, 60131 Ancona, Italy; a.marcelli@staff.univpm.it (A.M.); l.aquilanti@univpm.it (L.A.)

**Keywords:** vegetable waste, vegetable by-products, fermentation, high nutritional value, bioactive compounds, anti-nutritional factors

## Abstract

In recent decades, the rapid expansion of the food processing industry has led to significant losses and waste, with the fruit and vegetable sector among the most affected. According to the Food and Agriculture Organization of the United Nations (FAO), losses in this category can reach up to 60%. Vegetable waste includes edible parts discarded during processing, packaging, distribution, and consumption, often comprising by-products rich in bioactive compounds such as polyphenols, carotenoids, dietary fibers, vitamins, and enzymes. The underutilization of these resources constitutes both an economic drawback and an environmental and ethical concern. Current recovery practices, including their use in animal feed or bioenergy production, contribute to a circular economy but are often limited by high operational costs. In this context, fermentation has emerged as a promising, sustainable approach for converting vegetable by-products into value-added food ingredients. This process improves digestibility, reduces undesirable compounds, and introduces probiotics beneficial to human health. The present review examines how fermentation can improve the nutritional, sensory, and functional properties of plant-based foods. By presenting several case studies, it illustrates how fermentation can effectively valorize vegetable processing by-products, supporting the development of novel, health-promoting food products with improved technological qualities.

## 1. Introduction

**Figure 1:**
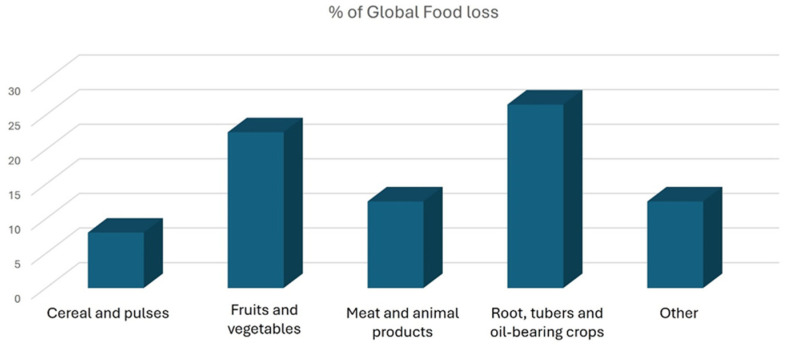
Food loss rates by commodity group along the post-harvest to distribution chain in 2016 [4].

In recent decades, the global food processing industry has expanded considerably, but this growth has also been accompanied by significant losses and waste, especially within the fruit and vegetable sector. As reported by the U.S. Environmental Protection Agency (EPA), in 2019 the food manufacturing and processing sectors in the USA produced 40.1 million tons of food waste [1]. Consistent with these findings, data collected in the United States by the United States Department of Agriculture (USDA) estimated that in 2010, over one-third of the food supply was wasted annually, with fruits and vegetables representing one of the most discarded categories, totaling 43.6 billion pounds [2,3]. Similar patterns have been observed on a global scale. According to the Food and Agriculture Organization of the United Nations (FAO), global food losses—from post-harvest through distribution—were estimated at 13.2% of total production in 2021, a figure that has remained relatively stable compared to previous years (13.0% in 2016 and 13.3% in 2020) [1]. Notably, fruits and vegetables consistently account for the highest loss rates among food categories, with estimates reaching as high as 60% [2,3]. An estimate of global food losses related to the main food categories is shown in Figure 1.

Food waste is typically considered to be edible material that is discarded despite being suitable for consumption. In line with this definition, vegetable waste refers to comestible parts of vegetables that are thrown away during post-harvest handling, packaging, distribution, and consumption [2]. These waste streams mainly comprise by-products like seeds, peels, rinds, stems, and pomace, which are valuable sources of bioactive compounds such as carotenoids, polyphenols, dietary fibers, vitamins, enzymes, and oils [2,5].

The vegetable processing industry generates millions of tons of these by-products which, despite their nutritional potential, are often discarded as waste in landfills. This results not only in the loss of edible materials but also in the inefficient use of key resources, including land, water, fertilizers, chemicals, energy, and labor. Such inefficiencies contribute to substantial economic losses, lowering farmers’ incomes and driving up consumer prices. From an environmental perspective, the disposal of food waste, particularly in landfills, contributes to severe ecological challenges, as its decomposition releases greenhouse gases, accelerating the degradation of natural ecosystems. Furthermore, addressing food waste has become imperative due to the increasing pressure on global resources driven by the inevitable rise in the world population [6,7].

The loss of horticultural products represents a global issue, with significant disparities between developing and developed countries, especially concerning the stages of the supply chain where these losses occur. In developing countries, losses are primarily attributed to inadequate infrastructure and handling practices, including deficiencies in storage, packaging, and marketing. Conversely, in developed countries and affluent sectors of developing nations, food waste is more prevalent, often resulting from consumer negligence or intentional disposal [2].

Existing technologies allow for the conversion of vegetable waste and by-products into animal feed, biofuels, or sources of bioactive compounds, such as antioxidants and antimicrobials, for use in food and feed fortification. To convert vegetable waste into biofuels, biorefinery methods such as anaerobic digestion (AD), incineration, pyrolysis, gasification, and hydrothermal carbonization are currently employed [3]. In the food industry, traditional and commercial utilization methods include microwave-assisted extraction (MAE), supercritical fluid extraction (SFE), ultrasound-assisted extraction (UAE), and high-pressure processing (HPP) [3].

However, to improve the environmental sustainability of vegetable processing, it is necessary to explore alternative solutions. Fermentation has demonstrated the potential to become a promising strategy for converting vegetable by-products into sources of high-value functional ingredients rich in substances such as antioxidants, vitamins, and prebiotics, which can be used to formulate innovative and widely consumed functional foods. Indeed, the fermentation of vegetable by-products can potentially improve the bioavailability of bioactive compounds, reduce anti-nutritional factors, and enhance the sensory characteristics of final food products [8].

Fermentation, though recognized as an efficient, affordable, and extensively utilized food-processing technique, can present health risks when ingredient quality, hygiene, and safety standards are not adequately maintained, particularly in small-scale operations and in low- and middle-income countries.

Inadequate food safety systems, combined with poor processing and storage practices, elevate the risk of contamination by pathogenic microorganisms.

Although foodborne illnesses from fermented foods are rarely reported, underreporting and weak surveillance may contribute to this. Pathogens such as *Escherichia coli*, *Salmonella* spp., *Listeria monocytogenes*, and *Bacillus cereus* have been detected in fermented foods, especially in Africa and Asia [9].

This review outlines the main aspects of fermentation as a sustainable method for converting vegetable by-products into high-value food ingredients. It also highlights the pivotal role of microorganisms and discusses future directions for improving fermentation efficiency. Additionally, recent studies on improving the nutritional profile of various vegetable by-products through fermentation by different microorganisms are reviewed and discussed.

## 2. Aim of the Review

The review seeks to offer a detailed overview of fermentation as an innovative and sustainable approach to exploit the potential of vegetable by-products generated by the food industry, highlighting the critical need to address global food waste.

By examining recent scientific advancements, the review explores how fermentation processes can transform vegetable by-products—often regarded as waste—into functional ingredients with significant nutritional and commercial value. Special attention is given to the influence of different microbial species on the fermentation of vegetables and their by-products from botanical families such as Solanaceae, Brassicaceae, and Poaceae.

The review underscores the dual role of fermentation in mitigating food waste and promoting the development of circular food systems that integrate environmental and economic sustainability while improving food quality and fostering product diversification.

## 3. Search Procedure

To gain a comprehensive overview of scientific research on the fermentation of vegetable by-products, a literature search was performed on the Scopus database “www.scopus.com” (accessed on 14 June 2024) utilizing the keywords “submerged fermentation”, “solid-state fermentation”, “vegetable waste and by-products”, “bioactive compounds”, “valorization”, “lactic acid bacteria”, “fungi”, “yeast”, “high nutritional value”, “sustainability” and “functional food” covering the period from January 2000 to June 2024. A total of 1009 records were found, covering two separate time intervals: period 1 (2000–2016), marked by a relatively constant number of published studies on vegetable waste and by-products fermentation, with a total of 37 hits, and period 2 (2016–2024), which exhibited a significant rise in research focused on vegetable waste and by-product fermentation, with a total of 975 hits in just 8 years.

The literature review was then limited to research articles addressing the production of valuable food products from vegetable waste and by-products through fermentation available in the PubMed “http://www.ncbi.nlm.nih.gov/pubmed” (accessed on 18 June 2024) and ScienceDirect “http://www.sciencedirect.com/” (accessed on 18 June 2024) databases, from 2000 to early 2024, using the following keywords: “vegetable by-product fermentation”, “submerged fermentation”, “solid-state fermentation”, “functional food”, “bioactive compound”, “anti-nutritional factor”, “lactic acid bacteria”, “fungi”, “yeast”, “high nutritional value”. A cross-referencing method was employed to identify additional relevant studies.

Articles were chosen based on the following inclusion criteria: (i) publication in a peer-reviewed journal, (ii) research related to fermentation of vegetable and vegetable by-products to obtain new ingredients with improved nutritional value, (iii) published in English, and (iv) published from 2000 to 2024, since most data on the production of valuable food products from vegetable by-products through fermentation were released during this interval. No limitations were imposed regarding the geographical origin of the vegetables. Documents such as proceedings, project files, theses, and articles unrelated to the production of food ingredients from vegetable waste and by-product fermentation were excluded.

### Database Generation

The information gathered from the 28 papers included: (i) publication details: authors, year of publication, and journal; (ii) fermenting microorganisms; (iii) fermentation method; (iv) vegetable family (i.e., leafy vegetables, stem vegetables, etc.) and species; (v) vegetable and vegetable by-product category; (vi) variation in the bioactive compound and anti-nutritional factor content in fermented vegetables and vegetable by-products; (vii) potential health properties of fermented products; and (viii) destination of use of the products obtained through vegetable and vegetable by-product fermentation.

## 4. Fermentation: A Sustainable Approach to Valorizing Vegetable By-Products

Fermentation is a biological process whereby organic macromolecules in the substrate are transformed into less complex, bio-functional, and nutrient-rich substances through the activity of enzymes and catalytic agents generated by microorganisms such as bacteria, yeast, and molds [10]. This longstanding method has been practiced for centuries to boost food sensory properties, for ritual feasts, and to extend food preservation during challenging seasons [8,10,11]. Historical records indicate its earliest use around 13,000 years ago for brewing beer from cereals [10,12].

Traditionally, fermentation depended on the indigenous microorganisms present in the food matrix, which were significantly influenced by the environment, resulting in the attributes of fermented foods also depending on the geographic area. However, with increasing global interest, consumption of fermented products, and heightened concern over food safety, it became necessary to standardize the method. This led to industrial oversight of the workflow, with the adoption of measures such as the use of starter and the application of controlled fermentation strategies on an extensive scale [10]. Today, fermentation is widely practiced owing to its well-documented advantages in prolonging storage stability and improving the sensory characteristics of fermented products [10]. The key advantage of fermentation lies in the ability of microorganisms to synthesize enzymes that degrade anti-nutritional factors, as highlighted by various authors [13,14]. This enzymatic activity not only increases the nutritional value and enhances digestibility but also imparts unique textures and flavors to the final products, making them more palatable [15,16] and increasing their commercial value [8]. Furthermore, fermentation also plays a role in supporting food safety by preventing the proliferation of pathogenic microorganisms [17].

Consequently, fermented products have become key ingredients in the development of novel foods across several sectors of the food industry, including food supplements, soup condiments, seasonings [18,19], infant food formulations [20], and cereal-based products like sourdough bread [8,21].

Fermentation not only enhances food properties but also offers a sustainable approach to repurposing food industry by-products into high-value ingredients. This is particularly relevant for the fruit and vegetable sector, which generates substantial processing waste [3,4,8].

Generally, there are four main types of food-producing fermentation (or fermentation applicable for food preservation): alcoholic, alkali, acetic, and lactic acid fermentation, classified based on the biochemical pathways involved [10]. Alcoholic fermentation occurs, for example, in beer and wine production and is usually driven by yeasts, that metabolize sugars in the substrate into ethanol and CO_2_ [10,22]. Conversely, bacteria predominantly from the *Acetobacter* genus carry out acetic acid fermentation by transforming alcohols and sugars into acetic acid; some well-known products of the acetic acid fermentation are vinegar and kombucha [10,23].

On the other hand, alkali fermentation is characterized by the hydrolysis of proteins into amino acids and peptides, and by the elevation of the pH (8–9) due to the release of ammonia, which inhibits spoilage-associated microbes and gives the final product a strong umami flavor and aroma. Alkali fermentation is mainly performed by *Bacillus* spp. and coagulase-negative staphylococci [10,24]. Lastly, lactic fermentation is usually performed by lactic acid bacteria, which are classified into two main physiological groups: homofermentative and heterofermentative, based on their fermentation pathway. The difference lies in the fermentation products. Homofermentative LAB perform homolactic fermentation, where sugars are mainly converted to lactic acid, while in heterofermentative fermentation, lactic acid bacteria transform sugars into lactic acid, CO_2_, acetic acid, and/or ethanol. Some examples of lactic acid fermentation products are yogurt and kimchi [10,22].

Lactic acid fermentation represents one of the most suitable, versatile, and promising approaches to converting vegetable by-products into new functional ingredients, whether it occurs naturally or is induced through starter inoculation [25,26].

Several authors have further investigated the benefits that lactic acid fermentation can bring to plant-based foods and, consequently, to consumers. Filannino et al. [25] and Tlais et al. [26] studied the metabolism of phenolic compounds during lactic acid fermentation, demonstrating the ability of this process to increase polyphenol bioavailability and stimulate the secretion of antioxidant enzymes, thereby contributing to radical scavenging potential. Particularly, lactic acid bacteria can hydrolyze phenolic acid esters and metabolize p-coumaric, ferulic, and caffeic acids into the corresponding reduced or vinyl derivatives, respectively, through the action of enzymes such as esterase, phenolic acid carboxylase, and reductase. This can improve the antioxidant properties of the substrate, as derivatives of lactic acid bacteria enzymatic activity could generate metabolites exhibiting greater biological functionality compared to their precursors [25,26].

Furthermore, the fermentation by lactic acid bacteria ascribed to the genera *Lactobacillus*, *Lactococcus*, *Leuconostoc*, and *Weissella* yields exopolysaccharides (EPS) that not only promote the growth of other probiotic bacteria but also offer immunomodulatory effects, antioxidant activity, and cancer prophylaxis. Additionally, the fermentation process releases bioactive peptides, contributing to functionalities such as blood pressure reduction, free radical scavenging, and antimicrobial activities [25,26]. Fermented plant matrices also exhibit increased contents of vitamins, short-chain fatty acids, and insoluble and soluble fibers [26,27].

The positive impact of LAB and yeast fermentations on the antiradical and antimicrobial activities of wasted plant materials also illustrates how these processes can boost the nutritional and health-promoting properties of plant products [26,28,29]. Examples include the use of carrot and tomato by-products in lactic acid fermentation, as well as the enrichment of bakery products with fermented olive mill by-products, demonstrating the feasibility of incorporating fermented by-product extracts into the food industry for both safety and nutritional enhancement [26,28]. Fermentation can be carried out using either solid-state (SSF) or submerged methods (SmF), as detailed in the following sections [8].

### 4.1. Solid-State Fermentation

Solid-state fermentation (SSF) is a biotechnological technique in which microorganisms grow on solid materials in the presence of a continuous gas phase. Although small amounts of water may exist between particles, SSF is characterized by minimal to no free water, with interstitial spaces occupied by gases that support microbial development [30]. The low water activity in SSF offers several advantages, including easier product recovery, lower production costs, simplified post-fermentation processing, and lower energy consumption for mixing and sterilization [30,31]. The process typically involves a moisture content ranging from 12% to 70%, with lower levels halting biological activity [8,32].

In addition to water activity, factors such as temperature, aeration, agitation, heat transfer, and pH play crucial roles in SSF, influencing microbial growth. Nutrient requirements, including macro- and micronutrients, are also critical for regulating and improving the metabolic activities of microorganisms during fermentation and are related to the fermented substrate. Optimizing these factors is necessary to achieve high productivity in SSF [33,34].

Molds are frequently employed in SSF because they naturally colonize solid substrates like wood, seeds, stems, and roots, and their growth is favored by low moisture conditions. Although bacteria and yeasts typically need higher moisture levels for optimal fermentation, they can still be employed in solid-state fermentation (SSF), though often with reduced yields. [31]. During SSF, substrate dehydration decreases water activity, thereby limiting microbial growth and reducing specific growth rates. Conversely, higher water activity values enhance the growth of both bacteria and fungi, especially under SSF conditions [33].

SSF can be used to produce various microbial products at both laboratory and industrial scales, ranging from enzymes and organic acids to bioethanol and antibiotics [35]. Various agricultural and industrial solid wastes, including those from the food, agriculture, detergent, paper, textiles, and animal feed industries, can serve as substrates for SSF. Commonly used substrates in SSF include cereal grains, legume seeds, lignocellulosic materials like straw and wood shavings, and an extensive variety of botanical and zoological substrates. These substrates are composed of polymeric compounds that exhibit limited or no solubility in water, providing a concentrated source of nutrients for microbial growth [30,31,36].

The SSF process typically includes substrate selection and optional pretreatment, followed by polymer hydrolysis, fermentation, and subsequent downstream steps for product recovery and analysis [31].

SSF offers significant advantages over SmF, such as higher product concentrations (with conversion rates of 20–30% compared to approximately 5% in SmF) [8,37], lower water consumption, reduced wastewater generation, lower water activity (which reduces susceptibility to contamination), easy gaseous transport, higher volumetric productivity with compact bioreactors, and simpler downstream processes [33,38,39].

Despite its benefits, SSF faces limitations in large-scale applications, particularly regarding process duration and heat management. Recent advancements, such as the mechanical index (Imp) proposed by Zhang et al. [40], provide a means for characterizing the physical properties of the medium and improving control over the fermentation process, offering promising ways to overcome these limitations [40,41].

The structural characteristics of the solid matrix, such as elasticity, cohesiveness, and resilience, collectively contribute to the parameter known as “Imp.” Throughout different stages of fermentation, Imp exhibits both positive and negative correlations with factors such as thermal conductivity, water retention, gas permeability, thermal diffusivity, and biomass content, all of which fluctuate during the fermentation process. Heat and mass transfer are directly influenced by these physical properties, ultimately influencing the efficiency of fermentation. Consequently, Imp serves as a valuable tool for assessing the physical traits of the substrate and enabling more precise control of solid-state cultures [41].

In summary, SSF offers several advantages over submerged fermentation methods, making it an attractive option for converting organic waste into functional and economically valuable outputs. The simplicity of the process, coupled with the use of abundant low-cost biomaterials, makes SSF a promising technology with significant potential in both laboratory and industrial applications.

### 4.2. Submerged Fermentation

Submerged fermentation (SmF) is widely used to cultivate microorganisms and has traditionally been applied to vegetables for preservation and nutritional enhancement. Some examples of fermented vegetables include sauerkraut [42,43], paocai [44,45], zhacai [45,46], kimchi [47], chili pepper [45,48], lotus root [45,49], carrot [45,50], ginger [45,51], cucumber [45,52], eggplant, beetroot [45,53], garlic [45,54,55], olive [45,56], papaya, and chayote [45,57], and sea fennel [58].

In this process, microorganisms grow uniformly in a liquid medium with constant agitation and aeration, promoting efficient substrate utilization and metabolite production [8,37].

SmF offers several advantages, including precise control over fermentation parameters such as temperature, moisture, and pH due to the homogenization of the liquid culture [8]. Additionally, it reduces the risk of moisture loss in fungal hyphae when molds are involved [8,36], has fewer limitations in scaling up processes [8], and allows a broader range of microorganisms to thrive [8,32]. The fully liquid environment of SmF is especially suitable for bacterial cultivation, as it meets their high-water activity needs [8]. A notable limitation of conventional SmF is the increased broth viscosity resulting from mold growth, which impairs mass transfer and oxygen diffusion [8,37].

**Figure 2:**
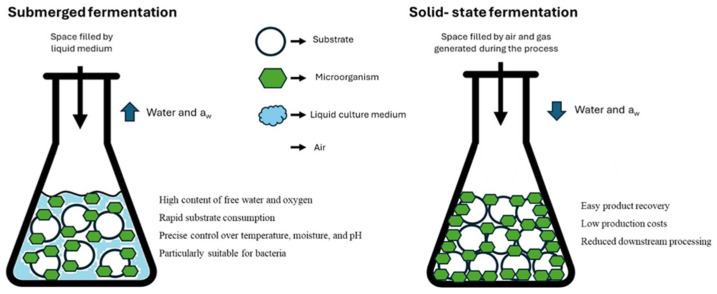
Comparison between submerged and solid-state fermentation.

SmF substrates vary widely, including simple sugars (glucose, lactose, sucrose) and cost-effective agro-industrial by-products like cheese whey, malt sprouts, and molasses [34,59,60]. The carbon/nitrogen ratio plays a crucial role in developing efficient nutrient media [34]. Differences between SmF and SSF fermentation are summarized in Figure 2.

Submerged fermentation is widely used in industrial settings for metabolite production due to its simpler downstream process and better control over fermentation parameters compared to solid-state fermentation. While SSF may be more suitable for the production of microbial secondary metabolites, it presents challenges in terms of operational parameters, making SmF the preferred option [33,61]. However, the choice between submerged and solid-state fermentation is mainly influenced by both the target product and the nature of the microorganism involved [8,34].

Different submerged fermentation techniques, including batch culture, fed-batch culture, continuous culture, and perfusion batch culture, offer various advantages and disadvantages depending on the end-use and microorganism type [62].

Batch culture, the simplest and most common method, involves inoculating microbes into a fixed volume of medium, allowing them to grow for a defined period, and then harvesting the culture. The chief limitation of this technique lies in the decreasing viability of microorganisms during the process due to both nutrient scarcity and the accumulation of harmful metabolites. Batch culture is commonly used in the initial stages of process refinement [62,63]. In contrast, the fed-batch mode involves periodic substrate addition to maintain ideal concentrations of essential nutrients throughout the process, while simultaneously carrying out continuous product harvesting [64,65]. The fed-batch approach has been successfully applied in the commercial production of biofertilizers, showing increased efficiency in terms of yield coefficient and productivity. Another method is the continuous culture, where the reactor sustains a steady inflow of medium at a set flow rate while simultaneously removing cells and supernatant. This maintains a constant reactor volume, enabling the maintenance of a specific growth rate for extended periods. Compared to batch reactors, fed-batch and continuous reactors provide higher viable cell counts and increased efficiency metrics, such as yield coefficient and production rate. However, a significant drawback of the continuous mode is the risk of washout when dilution rates exceed the growth rate [34,64,66]. An alternative, the perfusion reactor, involves recycling operations where cells are separated and retained within the reactor. This allows operation at dilution rates exceeding the growth rate while achieving higher cell densities compared to conventional continuous bioreactors. The various possible configurations of submerged fermentation demonstrate its versatility and widespread use in industry. However, optimization of fermentation parameters, medium components, and bioreactor design are essential for achieving high biomass production and metabolic activity [62,63].

Overall, submerged fermentation represents a flexible and well-controlled technique for microbial cultivation in liquid media, widely applied in the production of biomass, spores, and metabolites, as well as in vegetable fermentation. The choice of fermentation mode, nutrient media, and optimization strategies play key roles in achieving high biomass production and metabolic activity, making submerged fermentation a valuable tool in biotechnological processes [8,34].

## 5. Fermenting Microorganisms

Various fermenting microorganisms, such as both starter and non-starter lactic acid bacteria (LAB and NSLAB) and yeasts, are crucial agents in converting agri-food residues into valuable products by improving nutrient extraction and generating compounds of high commercial and nutritional value [67,68]. This chapter provides an in-depth comparison of these microorganisms, with a particular focus on LAB, and discusses their interactions and contributions to the fermentation of vegetable and fruit by-products.

### 5.1. Lactic Acid Bacteria

Lactic acid bacteria are a diverse group of Gram-positive, non-spore-forming microorganisms belonging primarily to the phyla Firmicutes and Actinobacteria. They are known for utilizing carbohydrates, particularly glucose, sucrose, and lactose, converting them into lactic acid, carbon dioxide, and other metabolites [69,70].

These microorganisms are typically grouped as thermophilic (40–50 °C), mesophilic (20–40 °C), and psychrophilic (0–20 °C) depending on their optimal growth temperature [71,72]. Metabolically, they are distinguished as either homofermentative or heterofermentative. Lactic acid bacteria utilize both homofermentative and heterofermentative metabolic routes, allowing them to effectively break down diverse carbohydrates, extending even to complex polysaccharides like cellulose and hemicellulose. Such metabolic versatility enables these bacteria to efficiently degrade the complex sugars present in vegetable waste, unlike other fermenting organisms that often depend on simpler sugars like glucose and may have limited substrate utilization [68,73]. Furthermore, lactic acid fermentation frequently yields advantageous metabolites—including vitamins, organic acids, bacteriocins, and flavor-enhancing compounds—that collectively improve the nutritional profile, health-promoting potential, and sensory characteristics of the fermented product [68].

This fermentation process generally takes place in anaerobic or low-oxygen environments, conditions which are highly conducive to the growth and activity of lactic acid bacteria. In contrast, alcoholic fermentation requires strict anaerobic conditions and specific temperature ranges, complicating the fermentation process and limiting the types of by-products that can be processed [10,68]. Moreover, lactic acid bacteria rapidly bring down the pH of the environment, leading to conditions that are adverse to spoilage microorganisms and pathogens.

Rapid acid production serves both to preserve the product and prolong its shelf life, positioning lactic acid fermentation as a favored technique over slower processes that risk contamination by spoilage organisms [68].

Therefore, lactic acid fermentation is widely employed in producing various fermented foods, including yogurt, sauerkraut, and pickles. The most notable genera of lactic acid bacteria, such as *Lactobacillus*, *Leuconostoc*, *Pediococcus*, and *Streptococcus*, are renowned for their roles in fermenting several substrates, including vegetables and fruits [22,68].

Commonly used species as starters in food fermentation, including vegetable fermentation, are *Leuconostoc mesenteroides*, *Lactiplantibacillus plantarum*, *Pediococcus acidilactici*, *Levilactobacillus brevis*, *Lactiplantibacillus pentosus*, *Lacticaseibacillus rhamnosus*, *Lacticaseibacillus casei*, *Lactococcus lactis*, *Latilactobacillus curvatus*, and *Lactobacillus delbrueckii* subsp. *bulgaricus* [74,75,76].

Some lactic acid bacteria also possess probiotic properties, promoting gut health when consumed. This aspect of lactic fermentation sets it apart from other fermentation methods, as the incorporation of probiotics adds additional health benefits to fermented vegetable products [68].

The genera *Lactobacillus*, *Lacticaseibacillus*, *Limosilactobacillu*, *Ligilactobacillus* and *Bifidobacterium* include the species most frequently employed as probiotics. The *Lactobacillus* species most utilized for probiotic purposes include *Lactobacillus acidophilus*, *L. plantarum*, *L. rhamnosus*, *Lacticaseibacillus paracasei*, *L. casei*, *Lactobacillus gasseri*, *Lactobacillus johnsonii*, *Limosilactobacillus reuteri*, *Limosilactobacillus fermentum*, *Ligilactobacillus salivarius*, and *L. delbrueckii* subsp. *bulgaricus*. Regarding the genus *Bifidobacterium*, the species most frequently utilized in food products include *Bifidobacterium adolescentis*, *Bifidobacterium animalis* subsp. *lactis*, *Bifidobacterium bifidum*, *Bifidobacterium breve*, *Bifidobacterium longum* subsp. *longum*, and *Bifidobacterium longum* subsp. *children* [74,76].

One of the most striking characteristics of lactic acid bacteria is their exceptional resilience to challenging environmental stresses. For instance, during the fermentation of onion by-products, lactic acid bacteria demonstrated significant growth and metabolic activity even in low pH conditions (pH ranging from 3.83 to 4.4). This robustness is linked to specialized metabolic mechanisms that enable LAB to sustain energy generation and essential cellular activities even under adverse conditions [25,77].

Throughout fermentation, lactic acid bacteria can also synthesize antimicrobial agents like bacteriocins that suppress pathogenic microbes, thereby enhancing the safety and shelf stability of the fermented products. These attributes make LAB particularly valuable in by-product valorization, where maintaining both the safety and quality of the product is essential [68].

In addition, lactic acid bacteria possess diverse enzymatic capabilities that enable them to break down complex polysaccharides and plant proteins, facilitating the liberation of phenolic compounds along the fermentation process. Enzymatic hydrolysis of plant cell walls by microbial enzymes, including amylases, cellulases, and xylanases, is crucial for degrading lignocellulosic matrices and releasing phenolics, thereby enabling efficient fermentation of fibrous vegetable residues [25,26].

Research has shown that the enzymatic functions of lactic acid bacteria are closely linked to increased phenolic bioavailability, highlighting their potential utility in fermenting vegetable and fruit waste [77].

For instance, Ramires et al. [77] reported that fermenting onion skins with *L. plantarum* substantially raised the levels of quercetin aglycone, a powerful antioxidant, by selectively converting glycosylated quercetin forms through targeted deglycosylation. This research indicates that lactic acid bacteria can effectively convert quercetin glycosides into aglycone forms during vegetable fermentation through their deglycosylation activities, enhancing the bioavailability of these beneficial compounds, improving their absorption in the human body, and making the fermented vegetables more appealing for consumption [77,78,79,80].

### 5.2. Fermenting Microorganisms Other than Lactic Acid Bacteria

Although lactic acid bacteria dominate the fermentation processes of vegetable and fruit by-products, other microorganisms, including yeasts, fungi, and members of the Proteobacteria phylum such as *Acetobacter* and *Bacillus* species, significantly contribute to these fermentations. These microbes participate in targeted fermentation activities, notably producing organic acids such as acetic and butyric acid, while efficiently breaking down proteins and carbohydrates found in vegetable by-products [81,82,83].

#### 5.2.1. Acetic Acid Bacteria

Acetic acid bacteria (AAB), including species within the genus *Acetobacter*, are strictly aerobic, Gram-negative bacteria belonging to the class Alphaproteobacteria, order Rhodospirillales, and family Acetobacteraceae [84]. These bacteria hold industrial significance, being extensively utilized for manufacturing vinegar (including fruit vinegars), gluconic acid, and in biofuel cell technologies [85,86,87]. AAB exhibit tolerance to elevated acetic acid levels and acidic environments; however, their optimal growth typically occurs within a pH range of 5.5 to 6.3 [88]. The ideal temperature range for AAB growth is 25–30 °C [82,88].

Key metabolic routes in AAB encompass the ethanol oxidation respiratory chain, the tricarboxylic acid cycle, pyruvate metabolism, and the pentose phosphate pathway. Among these, the aerobic fermentation pathway enables the partial oxidation of sugars, alcohols, or sugar alcohols, leading to the formation of aldehydes, ketones, and organic acids, including acetic acid [81,89]. These bacteria mainly convert yeast-derived ethanol into acetic acid, which not only intensifies the acidity and distinctive tartness of fermented products but also imparts antimicrobial effects. This oxidative process requires oxygen and is essential for flavor development in products like kombucha [82,88].

The acetic acid bacteria involved in acetic acid production are mainly classified within the genera *Acetobacter* and *Gluconobacter*. *Acetobacter aceti* is the best-known species, extensively utilized in vinegar production because of its efficient ethanol oxidation capacity, converting it into acetic acid. *Gluconobacter* species, such as *Gluconobacter oxydans*, also contribute to acetic acid synthesis but are particularly known for their role in partial oxidation reactions. These bacteria are essential contributors to both artisanal and large-scale vinegar manufacturing. Other species potentially involved in acetic acid production include *Acetobacter pasteurianus*, *Acetobacter nitrogenifigens*, *Gluconacetobacter saccharivorans*, *Gluconacetobacter diazotrophicus*, *Komagataeibacter europaeus*, and *Komagataeibacter xylinus* [85,90,91,92,93].

As a vital organic acid worldwide, acetic acid’s increasing demand has spurred research into environmentally sustainable production approaches. In the past few years, aerobic fermentation has gained attention as a method for acetic acid production using food waste [82,90].

A broad spectrum of substrates, including cereals, fruits, vegetables, and by-products such as cloudberries, strawberries, persimmons, onions, sweet potatoes, and kitchen scraps, serve as feedstock for acetic acid fermentation, yielding nutrient-dense and flavorful vinegars. Patents document innovative methods for producing unique vinegars, such as cloudberry vinegar (with *Acetobacter aceti*), 100% onion vinegar, and sweet potato vinegar with tea, as well as an efficient two-step kitchen waste fermentation process involving yeasts and AAB. Additionally, molasses and brown sugar have been evaluated as substrates [82]. Consequently, AAB are key contributors in valorizing vegetable by-products via aerobic fermentation, offering a dual benefit of minimizing food waste and fostering sustainable production by transforming underutilized materials into valuable commodities.

#### 5.2.2. Other Bacteria

Several other fermentative bacteria have been investigated for their potential in vegetable by-product valorization. Notably, *Bacillus* and *Clostridium* species stand out for synthesizing functional ingredients and bioactive molecules, including lactic acid, poly-γ-glutamic acid, glycosidases, and caproate from vegetable-derived substrates. Studies have shown diverse applications for these bacteria, particularly in producing ingredients suitable for reintegration into the food processing system [83].

*Bacillus* species are recognized chiefly for their enzymatic repertoire, including protease and cellulase activities, which aid in decomposing complex organic compounds and improving fermentation performance. Such enzymatic processes promote the liberation of amino acids and peptides, consequently enhancing the nutritional quality of the fermented outputs. *Bacilli* are also able to produce enzymes that degrade polysaccharides, a skill particularly beneficial in the fermentation of lignocellulosic substrates, as it allows for better nutrient recovery from complex materials. Within the *Bacillus* genus, species such as *Bacillus coagulans*, *Bacillus licheniformis*, and *Bacillus subtilis* are frequently employed to ferment various by-products from rice, soy, oats, fruit, and sorghum [67,68,83,94,95,96].

For example, *B. coagulans* is employed in the conversion of rice bran into high-purity L-lactic acid (>99%) after enzymatic hydrolysis, achieving a 90% conversion yield with suitable strains [83,94].

In addition to rice bran, soybean by-products such as dregs, meat, and okara are also valuable substrates for fermentation. A study carried out by Mukherjee et al. [96] showed that soybean meal, when fermented by a mixed culture of *B. coagulans* and *L. johnsonii*, displayed improved antioxidant properties [83,96].

*Clostridium* species, being anaerobic bacteria, are particularly notable for fermenting fibrous vegetable and fruit residues, converting lignocellulosic biomass into various value-added compounds, frequently applied beyond the food sector [67,83].

An example involves *Clostridium cellulovorans* and *Clostridium beijerinckii* strains fermenting mandarin orange by-products. Typically, D-limonene, a compound present in citrus fruits, poses challenges for fermentation by inhibiting yeast activity and making ethanolic fermentation difficult. However, the growth of these strains remains unchanged in the presence of physiological amounts of D-limonene. This adaptation enables biofuel production from citrus by-products, leveraging the isopropanol–butanol–ethanol fermentation capability of *C. beijerinckii* and the capacity of *C. cellulovorans* to degrade cellulosic biomass [83,97].

In summary, *Bacillus* and *Clostridium* species represent promising agents for fermenting vegetable by-products, producing valuable compounds including functional food ingredients and additives [83].

#### 5.2.3. Yeasts

Yeasts are eukaryotic microbes inhabiting diverse ecological environments such as water, soil, air, and notably the surfaces of plants and fruits. The fruit surface environment is especially important, as yeasts play an active role in breaking down ripe fruit and facilitating spontaneous fermentation. In this environment, they obtain the necessary nutrients and substrates to sustain their metabolism and fermentation activities [98].

Metabolically, yeasts possess the capacity to ferment various sugars, mainly glucose, fructose, sucrose, maltose, and maltotriose, which are commonly present in ripe fruits and processed cereal products. This makes them suitable for converting agri-food by-products into valuable products [98]. For instance, fermentation of olive-mill wastewater by *Yarrowia lipolytica* can produce citric and oleic acid [83,99], while *Saccharomyces cerevisiae* can enhance the nutritional properties of potato flour through fermentation [100].

Yeasts also exhibit acid tolerance, surviving at pH levels near or below 3.5. Technologically, they are broadly classified into *Saccharomyces* and non-*Saccharomyces* genera [98].

Yeasts are well known for their central role in alcoholic fermentation, which underpins the consistent quality of fermented products such as alcoholic drinks and bread [98,101]. The genera commonly involved in fermentation, such as *Saccharomyces*, *Candida*, *Pichia*, *Kluyveromyces*, and *Debaryomyces*, primarily metabolize sugars (e.g., glucose and fructose) to produce ethanol and carbon dioxide [68].

Under anaerobic conditions, yeasts ferment glucose through glycolysis to yield ethanol and carbon dioxide. This biochemical pathway not only imparts unique flavors and aromas to fermented products but also produces ethanol, which acts as a preservative by suppressing spoilage microbes. However, this reaction is suboptimal for yeast cells, requiring them to consume large amounts of glucose to produce adequate ATP, leading to ethanol accumulation. Once this occurs, fermentative activity halts [10,98,101].

Yeasts also generate various volatile metabolites, such as esters and higher alcohols, which enrich the sensory profile of fermented foods by adding fruity and floral nuances. The addition of precursors like leucine and valine can result in the production of isoamyl acetate, a compound with a banana-like aroma, enriching the sensory profile of fermented foods [73].

In co-culture with lactic acid bacteria (LAB), yeasts engage in synergistic fermentations that enhance both flavor complexity and nutritional attributes. Kombucha is a notable example, where interactions between yeasts and LAB contribute to complex flavors and health benefits. A balanced relationship between yeasts and LAB is essential for successful fermentation, with each group playing distinct roles at various stages [10].

Additionally, certain yeast strains exhibit enzymatic activities that complement those of LAB, such as the degradation of phenolic compounds, which diminishes bitterness and enhances palatability in fermented foods. Moreover, certain species—such as *Zygosaccharomyces mrakii*, *Candida boidinii*, and *Metschnikowia pulcherrima*—exhibit tolerance to low pH and high phenolic concentrations, making them well-suited for co-fermentation in vegetable and fruit by-products. However, these yeasts often differ from LAB in the types of by-products produced and the metabolic pathways they employ [77].

Furthermore, metabolites generated by yeasts, including ethanol, provide substrates for acetic acid bacteria (AAB), while LAB benefit from sugars liberated by yeast enzymatic activity, fostering a complex and interdependent fermentation ecosystem [10].

In conclusion, yeasts are indispensable biocatalysts for converting renewable substrates into high-value bioproducts via fermentation. Fruit and vegetable by-products hold substantial potential as substrate sources due to their rich nutritional profile. Moreover, utilizing them as substrates supports the growing global commitment to a zero-waste policy. Some natural bioproducts generated by yeasts from vegetable and fruit by-product bioprocessing include enzymes, color and flavor agents, biopolymers, single-cell proteins, and biofuels—products that are widely applied across the food, chemical, biomedical, and other industries [102].

#### 5.2.4. Filamentous Fungi

Filamentous fungi excel in bioconversion applications using solid-state fermentation (SSF), thanks to their capacity to grow on substrates with limited moisture. This trait confers advantages over submerged fermentation (SmF), such as lower energy demands and increased process efficiency. While fungal SSF has a long history in East Asian food production, its adoption has broadened worldwide in recent years due to its effectiveness in producing valuable organic compounds and transforming agri-food residues into useful products, thus contributing to environmental sustainability. Industrial outputs of fungal SSF encompass enzymes, organic acids, biofuels, and diverse bioactive metabolites—including antibiotics, pigments, and biocontrol agents—serving industries such as food, feed, pharmaceuticals, cosmetics, bioenergy, and agriculture [103].

Examples of enzymes produced via fungal fermentation include xylanase from brewer’s spent grain [104], phytase from triticale waste [105], and α-amylase derived from starchy by-products such as soybean and wheat bran [106,107], protease from soybeans and wheat bran [108], and lipase from lipid-rich agro-by-products like olive pomace [109].

Additionally, molds contribute to the flavor complexity of fermented foods by synthesizing secondary metabolites, a key factor in the manufacture of traditional products like tempeh, miso, and soy sauce. Molds can also facilitate the conversion of agricultural by-products into bioethanol and other bio-based products, as well as novel fermented food products [31,67,68].

For example, cellulose-rich by-products fermented by xylanolytic fungi, such as *Aspergillus* and *Trichoderma* species, yield prebiotic substrates like arabinoxylo-oligosaccharides and xylose from materials such as brewer’s spent grain, rice husks, soybean hulls, and grape pomaces [110,111,112]. *Aspergillus* spp. can also produce fructo-oligosaccharides through the fermentation of agave aguamiel, sugarcane bagasse, and banana by-products [113,114], while isomaltulose, a prebiotic sweetener, is derived from cane molasses [50,83].

Adapted for SSF, filamentous fungi produce a wide spectrum of extracellular enzymes and utilize specialized secretion mechanisms that enable nutrient acquisition and survival on lignocellulosic materials with low moisture content [115]. These fungi produce a variety of hydrolytic enzymes, such as cellulases, xylanases, and amylases, that break down complex carbohydrates in vegetable by-products. This enzymatic activity releases simple sugars and phenolic compounds, which can be utilized by other microorganisms in subsequent fermentation stages. The filamentous fungi most employed in vegetable by-product fermentation, particularly via SSF processes, belong to genera such as *Aspergillus*, *Mucor*, and *Rhizopus* [68].

Combining SSF and SmF techniques can optimize the valorization of agricultural residues, leading to increased fungal biomass production. For example, Gmoser et al. [116] converted ethanol plant residues, such as ”thin stillage,“ and waste bread into raw materials for producing extra ethanol, fungal biomass, and a protein-enriched feed product by exploiting the fungus *Neurospora intermedia*—first in submerged fermentation, followed by solid-state fermentation. Similarly, Intasit et al. [117] achieved a 4.2-fold increase in carbohydrase production through a sequential co-culture of *Aspergillus tubingensis* and *Trichoderma reesei* on palm empty fruit bunches (EFB) using combined SSF and SmF processes [115,117].

Thus, the diverse metabolic capabilities of fungal species provide an excellent foundation for developing fermentation strategies to sustainably maximize the production of value-added ingredients from vegetable residues [83].

## 6. Fermented Vegetable By-Products

**Figure 3:**
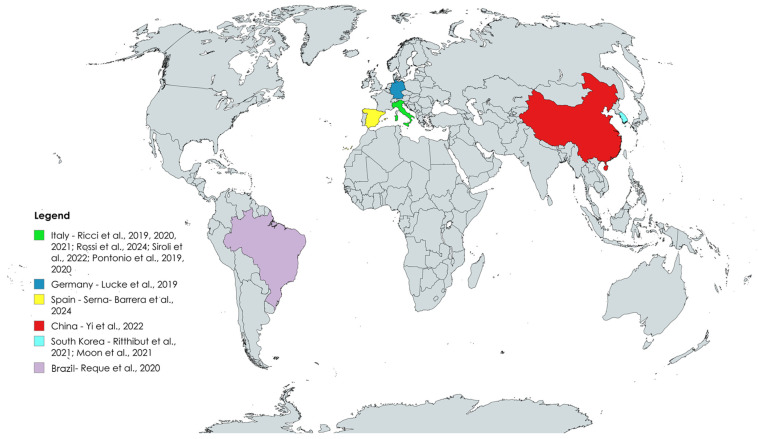
Geographical distribution of vegetable by-product fermentation case studies. Author’s elaboration based on data reported in Ricci et al. [28,118,119]; Rossi et al. [124]; Siroli et al. [125]; Pontonio et al. [122,126]; Lucke et al. [121]; Serna-Barrera et al. [120]; Yi et al. [129]; Ritthibut et al. [127]; Moon et al. [128] and Reque et al. [123].

The case studies discussed in the following chapters are listed in Table 1. Their geographical distribution is shown in Figure 3.

**Table 1:** Case studies on fermentation of vegetable by-products performed by lactic acid bacteria, yeasts and filamentous fungi.

Target Vegetable	Microorganisms	Fermentation Parameters	Fermented Substrate	Results	Reference
Solanaceae					
Tomato	*L. plantarum*, *L. casei*, *L. paracasei* and *L. rhamnosus*	Submerged (30 °C for *L. plantarum*, 37 °C for *L. casei*, *L. paracasei* and *L. rhamnosus*—72 h)	By-product extract	Increased antimicrobial activity against *Listeria monocytogenes*, *Salmonella* spp., *Escherichia coli*, *Staphylococcus aureus* and *Bacillus cereus*.	[28]
	*L. plantarum*, *L. casei*, *L. paracasei* and *L. rhamnosus*	Submerged (30 °C for *L. plantarum*, 37 °C for *L. casei*, *L. paracasei* and *L. rhamnosus*—72 h)	By-product extract	Increased antimicrobial activity in vitro and in foodstuff, surpassing those of commercial preservatives against spoilage microorganisms and foodborne pathogens such as *Salmonella* spp., *L. monocytogenes*, and *B. cereus*.	[118]
	*Pediococcus acidilactici*, *L. rhamnosus*	Submerged (37 °C for 6/14/24/30/48 h)	Tomato puree, tomato var. ciliegino juice and tomato var. pizzutello juice	Increased antioxidant activity and total phenolic content (except for tomato puree).	[119]
Brassicaceae					
Broccoli	*L. reuteri*, *L. plantarum* and *L. salivarius*	Submerged (37 °C for 7/24/48/72/96 h)	Stem powder	Increased total phenols and flavonoids.	[120]
Rapeseed	*Rhizopus microsporus* var. *oligosporus*	Solid state fermentation (32 °C for 48 h)	Rapeseed presscake	Decreased anti-nutritional factors, glucosinolates, cell wall polysaccharides and phenolic compounds.	[121]
Poaceae					
Maize	*L. plantarum T6B10* and *Weissella confusa BAN8*	Submerged (30 °C 24 h)	Milling by-products	Increased free amino acids and peptides concentrations. Enhanced antioxidant activity and total phenol content. Decreased phytic acid, lipase activity (preventing oxidative processes). Increased nutritional, textural and sensory properties of wheat bread. Increased protein digestibility in fortified bread. Relevant lowering of starch hydrolysis index in fortified bread.	[122]
Wheat	*L. plantarum DSM 20174T*, *Lactiplantibacillus fabifermentans T30PCM38*, *L. fermentum LM7*, and *Streptococcus thermophilus TH985*	Submerged (37 °C for 24 h)	Wheat middlings	Improved total phenolic content, antioxidant, anti-browning, antibacterial, and prebiotic properties.	[123]
	*L. curvatus*, *L. mesenteroides*, *Pediococcus pentosaceus*, *Kazachstania servazzii Kazachstania unispora*	Solid state fermentation (25 °C for 24 h)	durum wheat by-products (micronized bran and middling	Increased alcohols, ketones, acids, peptides, short-chain fatty acids. Increased total phenols, antioxidant activity and prebiotic activity. Decreased phytic acid content.	[124]
	Consortium composed of *Kazachstania unispora*, *Kazachstania servazii* and *L. curvatus*	Submerged (25 °C for 24 h)	milling by-product mixture (70% rye bran and 30% wheat germ)	Increased complexity of aroma-related compounds such as acids, alcohols and esters. Increased short-chain fatty acids, antioxidant activity, total phenol content, bioactive peptides, prebiotic activity. Decreased phytic acid content. Enhanced color stability compared to fermentation with baker’s yeast.	[125]
Spring hull-less barley, emmer, blue- and yellow-grained wheat varieties and one conventional redgrained wheat variety	*L. plantarum* and *Weissella confusa*	Solid state fermentation (30 °C for 24 h)	brans from wheat, barley and emmer	Increased radical scavenging activity, TFAA and phenolic compounds, free amino acids concentration and peptides. Enhanced in vitro protein digestibility. Decreased phytic acid content. Increased dietary fibers, proteins and protein digestibility in fortified bread. All fortified breads showed improved nutritional value.	[126]
Rice	*Aspergillus brasiliensis*, *Aspergillus awamori*, *Aspergillus sojae*	Solid state fermentation (25 °C for 8 days (*Aspergillus brasiliensis*) or 14 days (*Aspergillus awamori*, *Aspergillus sojae*)	Rice bran	Enhanced antioxidant, cosmeceutical activities (tyrosinase and elastase inhibitory activities). Improved overall content of bioactive components.	[127]
	*L. plantarum*	Submerged (30 °C for 48 h)	Rice bran	Decreased cholesterol. Increased antimicrobial activities against foodborne pathogenic bacteria and food spoilage fungi. Decreased phytic acid.	[128]
	*L. plantarum*	Submerged (36 °C for 16 h)	Rice bran	Enhanced cooking qualities, sensory evaluation, and textural properties. Decreased aldehydes. Increased alcohols. Improved DPPH radical scavenging activity. Increased antioxidant bioavailability.	[129]

### 6.1. Solanaceae

Tomato (*Solanum lycopersicum* L.) ranks among the most widely consumed vegetables of the Solanaceae family [28,130].

As a key component of the Mediterranean diet [28], tomato is a valuable source of health-promoting compounds, offering a balanced profile of minerals and antioxidants such as vitamins C and E, lycopene, β-carotene, lutein, and flavonoids like quercetin. Research has linked tomato consumption with a reduced risk of certain cancers, cardiovascular diseases, and age-related macular degeneration [119]. More than 80% of tomatoes are consumed as processed products, leading to considerable sustainability implications for the agro-industrial sector regarding the management of the resulting by-products. Tomato by-products are rich in bioactive compounds, including polyphenols, carotenoids, and vitamins, which exhibit various physiological benefits [28].

An investigation by Ricci et al. [28,118] examined the antimicrobial properties of extracts obtained from tomato by-products fermented with *L. plantarum*, *L. casei*, *L. paracasei*, and *L. rhamnosus*, observing antimicrobial activity—which was practically absent before fermentation—against pathogenic strains of *Listeria monocytogenes*, *Salmonella* spp., *Escherichia coli*, *Staphylococcus aureus*, and *Bacillus cereus*. The highest antimicrobial activity was recorded against *Salmonella* spp., *L. monocytogenes*, *S. aureus*, and *B. cereus*, while lower activity was observed against *E. coli* [28]. Subsequently, Ricci et al. [118] evaluated the application of fermented tomato by-product extracts as a preservative in minced pork meat, reporting promising outcomes. Samples treated with the extract maintained a lower total microbial load compared to the control (untreated minced pork meat), and its effectiveness at concentrations of 1.6% and 2.4% was comparable to that of sodium lactate/sodium diacetate, a commonly used preservative in meat preservation [118,131]. Specifically, the results from a microbial challenge test performed on minced pork meat containing fermented tomato by-product extract at 1.6% concentration showed that the growth of *Salmonella* and *L. monocytogenes* was suppressed in the presence of the extract. It was noted that the antimicrobial effect did not correlate directly with LAB growth or pH reduction but was attributed to the combined action of fermentation-derived metabolites. Therefore, the observed antimicrobial activity may be linked to the capacity of LAB to produce antimicrobial compounds such as organic acids, hydrogen peroxide, carbon dioxide, bacteriocins, and phenolic substances during the fermentation process. LAB can also produce phenyllactic acids, which have documented antimicrobial effects. Given their capacity to prolong shelf life, fermented tomato extracts hold potential as natural preservatives, improving both the safety and stability of diverse food products. This aligns with consumer demand for reducing or replacing the use of artificial preservatives, which are often viewed as “chemicals” [28,118]. Furthermore, Ricci et al. [119] explored the potential of lactic fermentation to develop fermented tomato-based drinks with the presence of viable cells and potentially bioactive metabolites. For this purpose, one tomato puree and two tomato juices, produced respectively from *ciliegino* and *pizzutello* varieties, underwent submerged fermentation using *Pediococcus acidilactici* and *L. rhamnosus* strains. Results indicated substantial microbial growth, alongside increased antioxidant activity and total phenolic content in both tomato juices, whereas the puree proved unsuitable for lactic fermentation, exhibiting no such improvements [119]. These findings underscore the potential of lactic fermentation for developing tomato-based beverages enriched with bioactive compounds, while also emphasizing that fermentation outcomes are strongly influenced by the characteristics of the substrate [119].

Collectively, research by Ricci et al. [28,118,119] suggests that implementing fermentation for tomato by-products can help reduce global vegetable waste and create new, healthy, and sustainable food ingredients.

### 6.2. Brassicaceae

Several studies have demonstrated that fermentation can improve the nutritional properties of vegetables from the Brassicaceae family. Broccoli (*Brassica oleracea* var. *italica*), for instance, is a significant source of fiber, vitamins, minerals, and particularly phenolic compounds, which exhibit health-promoting properties such as free radical scavenging and inhibition of low-density lipoprotein oxidation in humans. It is also rich in glucosinolates (GLSs), isothiocyanates (ITCs), and indoles, natural bioactive metabolites linked to antioxidant, anticancer, and immunosuppressive effects, which occur in several cruciferous vegetables. To exert their biological effects, compounds such as GLSs must be hydrolyzed by the enzyme myrosinase, generating bioactive metabolites like ITCs and indoles [132]. Fermentation promotes the hydrolysis of phenolics and GLSs through microbial enzymatic activity, leading to the formation of health-beneficial metabolites [132].

Although rapeseed (*Brassica napus* subsp. *Napus*) is part of the Brassicaceae family, its consumption in the human diet is largely limited to its oil.

Currently, rapeseed products, such as rapeseed presscakes, are primarily utilized in animal feed or outside the food and feed industries. However, rapeseed protein possesses a favorable amino acid composition, high biological value, and notable digestibility, making it a promising candidate for human nutrition [121]. Nevertheless, rapeseed meal and presscakes contain undesirable compounds that reduce their nutritional value and affect sensory qualities. These include oligosaccharides like stachyose and raffinose, which can cause flatulence, and phytates, which interfere with mineral absorption. Fungal fermentation represents a valuable strategy to improve rapeseed meal by reducing anti-nutritional factors and secondary metabolites, while simultaneously enriching the substrate with proteins, vitamins, and other bioactive compounds [121].

Serna-Barrera et al. [120] studied lactic acid bacteria fermentation using *L. reuteri*, *L. plantarum*, and *L. salivarius* as a pretreatment to boost the antioxidant and probiotic properties of broccoli stem powders. Despite accounting for approximately 38% of the plant’s total weight, broccoli stems are frequently discarded during the production of minimally processed or ready-to-eat products, although they are rich in antioxidants, vitamins, fiber, carotenoids, phenolics, and glucosinolates. Consequently, these by-products possess valuable nutritional properties that could be harnessed for the development of novel functional ingredients.

Lactic acid bacteria fermentation has proven to be a promising pretreatment method for creating nutrient-enriched powders from broccoli stems. After 24 h of fermentation, there was a significant increase in total phenols (4.9 mg GAE/g_db_ to 15.2 mg GAE/g_db_) and flavonoid content (0.33 mg QE/g_db_ to 0.8 mg QE/g_db_). The increase in phenolic compounds was primarily driven by microbial enzymatic activity, particularly cellulases and glycosidases produced by lactic acid bacteria, which participate in the breakdown of plant cell walls. In addition to facilitating the liberation of bound phenolics, microbial metabolism also promoted the biosynthesis of novel phenolic compounds. This process may be further enhanced in finely ground samples, where a reduced particle size increases the surface area available for enzymatic action, thereby intensifying phenolic release and transformation [120].

Conversely, Lücke et al. [121] investigated the application of solid-state fermentation with the tempeh mold *Rhizopus microsporus* var. *oligosporus* to render rapeseed press cake suitable for human consumption. The results showed that solid-state fermentation can effectively reduce the levels of certain undesirable constituents in rapeseed press cake, including the degradation of glucosinolates, cell wall polysaccharides, and phenolic compounds. The reduction of glucosinolates following fermentation enhanced the nutritional quality of rapeseed press cake, as their hydrolysis products, particularly isothiocyanates, are known for their antioxidant and antiproliferative properties, contributing to potential health benefits [132]. However, to use the resulting product for protein enrichment in foods or as a protein substitute, the degradation level of undesirable compounds should be further standardized, especially by controlling pH, oxygen supply, and fermentation duration [121].

In summary, the findings of these studies suggest that fermented Brassicaceae by-products could contribute to waste reduction while serving as promising ingredients for the formulation of innovative functional foods, highlighting the versatility of lactic fermentation.

### 6.3. Poaceae

Global cereal production is projected to reach approximately 2853 million tons, in 2024, positioning cereals as a primary source of food worldwide [133]. The most widely consumed cereals belong to the Poaceae family, including wheat (*Triticum aestivum* L.), rice (*Oryza sativa* L.), corn (*Zea mays* L.), barley (*Hordeum vulgare* L.), rye (*Secale cereale* L.), oats (*Avena sativa* L.), and others [125,134]. The milling of these cereals produces by-products such as bran and germ, which are commonly used in animal feed but remain underutilized in human nutrition, despite being rich in fiber and bioactive compounds [125,135]. These by-products, if incorporated into bread and other food products, could enhance their nutritional profile [136].

However, their inclusion in food products can affect texture, flavor, and overall sensory quality. Due to its high fiber content, bran can disrupt the gluten network in dough, resulting in suboptimal bread texture and flavor. Similarly, enzymes present in germs may compromise the sensory stability of final products [122,137]. For example, rye bran contains significant amounts of cellulose, hemicellulose, and lignin, as well as bioactive compounds like ferulic acid, known for its antioxidant and anti-inflammatory properties [138]. Although rye bran offers notable health benefits, its application in food products remains restricted because of its adverse effects on sensory characteristics, which reduce consumer acceptability [139].

Fermentation represents a promising strategy for enhancing the functional properties of cereal bran by-products. Through the use of microbial strains such as lactic acid bacteria (LAB) and yeasts, fermentation can lower the concentration of anti-nutritional factors, boost nutrient uptake, and increase antioxidant activity alongside other beneficial effects [25,125]. For instance, fermentation by *L. plantarum* and *Weissella confusa* on maize milling by-products improved their nutritional properties, increased total phenol (1.6 mmol/kg to 1.8 mmol/kg), free amino acids (1431 mg/kg to 1905 mg/kg), and peptides (38.4 mg/g to 46.5 mg/g), and reduced phytic acid content (1.10 g/100 g to 0.53 g/100 g), ultimately enhancing bread quality. According to the authors, the observed rise in amino acid levels resulted from the combined proteolytic activity of sourdough LAB and endogenous proteases, which become activated under the acidic conditions established during fermentation—a finding consistent with previous research. LAB fermentation also created an optimal environment for phytase activity, resulting in a reduction of phytic acid levels. These proteolytic processes, driven by both endogenous enzymes and microbial peptidases, likely promoted the release of antioxidant peptides, thereby enhancing the overall radical scavenging capacity [122].

Multiple studies have investigated the fermentation of wheat by-products using various starter cultures to improve their nutritional quality. Reque et al. [123] examined fermentation of wheat middlings by lactic acid bacteria strains, observing enhanced antioxidants (400 µM Trolox equivalents/mL in DPPH assay and 600 µM Trolox equivalents/mL in FRAP assay) and prebiotic properties due to a rise in total phenolic content. The observed difference might have resulted from the release and increased solubility of phenolic compounds due to cell and granule disruption. LAB belonging to *Lactiplantibacillus* genus, including *L. plantarum*, are known to degrade phenolic compounds through enzymatic pathways. *L. plantarum* specifically metabolizes phenolic acids like coumaric, caffeic, ferulic, gallic, and protocatechuic acids via reductases and decarboxylases, with a strain-specific bioconversion [123]. Similarly, Rossi et al. [124] used a mixed culture of lactic acid bacteria and yeasts to ferment durum wheat by-products, leading to increased levels of peptides, short-chain fatty acids (SCFAs), and total phenolic compounds, as well as improved antioxidant activity. Additionally, this process lowered phytic acid levels and promoted the proliferation of beneficial probiotic strains, suggesting the potential of fermented wheat by-products as valuable ingredients for improving the nutritional and sensory properties of bread and pasta.

Fermentation increased bioactive peptide content through the proteolytic activity of LAB and *Kazachstania* spp. LAB, in particular, are well recognized for their ability to break down proteins and their potential to enhance the bioactive peptide content in various fermented foods, including milk, cereals, soy, and amaranth.

The fermentation process also resulted in an increase in short-chain fatty acids (SCFAs), particularly acetic and hexanoic acids, due to LAB metabolism [124]. Lactobacilli produce SCFAs by fermenting carbohydrate end products, such as pyruvate, generated through glycolysis and the phosphoketolase pathway under heterofermentative conditions [125]. The rise in SCFA concentrations is noteworthy given their established health benefits, which include modulating insulin secretion, inflammation, lipid metabolism, and gut health, alongside their role in enhancing bread crumb flavor during baking. Furthermore, LAB fermentation promoted the synthesis of exopolysaccharides and glutathione, boosting antioxidant activity by scavenging free radicals, thereby improving the health-promoting and functional aspects of the fermented durum wheat by-products [124]. Moreover, fermenting combinations of rye bran and wheat germ by a microbial consortium led to notable increases in short-chain fatty acids (more than 5-fold), antioxidant activity (+24%), and prebiotic properties, along with a reduction in phytic acid (−28%) [125]. The use of such fermented by-products in breadmaking resulted in higher protein digestibility (+40%), increased phenolic compounds (2.55 ± 0.03 mmol/kg to 4.23 ± 0.05 mmol/kg), and lower glycemic index compared to traditional bread. The decrease in the glycemic index was due to the significant contribution of dietary fiber and biological acidification from the inclusion of fermented bran in the bread formula, which reduced the rate of starch hydrolysis [126].

Beyond maize and wheat, other cereal by-products like rice bran have also been fermented to enhance their functionality. Studies by Ritthibut et al. [127] and Yi et al. [129] on rice bran fermentation showed increased antioxidant properties, improved sensory quality, and a reduction in phytic acid content, demonstrating the potential of fermented rice bran in functional food applications. Rice bran fermented with *L. plantarum* exhibited improved texture, better radical scavenging activity, and a reduction in cholesterol (45–68%), aldehydes, and phytic acid, leading to a more pleasant flavor profile [128,129]. The observed textural changes likely resulted from bran fermentation, which disrupted starch crystallites and granules, promoting swelling and molecular leaching within the rice bran matrix [129]. Cholesterol reduction in fermented rice bran was attributed to *L. plantarum*’s strong cholesterol-binding capacity, its ability to enhance bioactive compounds such as tocotrienols, γ-oryzanol, and dietary fiber, and the possible production of unidentified fermentation-derived compounds that further contributed to cholesterol reduction [128]. Additionally, aldehyde reduction may have occurred due to *L. plantarum*’s ability to convert aldehydes into alcohols or oxidize them to organic acids, increasing alcohol content and contributing to the development of more pleasant flavors [129]. Taken together, these findings indicate that fermentation with carefully selected microbial consortia can substantially improve the nutritional value and functional attributes of cereal milling by-products.

This approach provides an effective strategy for transforming cereal by-products into high-value food ingredients suitable for the bakery sector and other food and nutraceutical applications.

By improving the sensory qualities and nutritional value of cereal by-products, fermentation could foster the development of more sustainable and nutritious food products.

## 7. Future Perspectives in Fermentation

Future research on vegetable fermentation is expected to be increasingly guided by multi-omics strategies, combining transcriptomics, proteomics, and metabolomics to achieve a holistic view of microbial metabolism and its influence on fermentation processes. Transcriptomics analyzes gene expression to identify key metabolic pathways and regulatory mechanisms, providing insights into microbial adaptation and functional traits. Proteomics investigates the structure, function, and interactions of proteins, helping to elucidate enzyme activity and microbial responses during fermentation. Metabolomics, which examines the complete set of small-molecule metabolites, offers a direct representation of biochemical processes and fermentation-derived compounds [140]. Such methodologies are especially useful for mapping fermentation-related metabolic pathways and selecting lactic acid bacteria (LAB) strains suited to specific environments or capable of modulating phenolic compound profiles. However, integrating these complex datasets remains challenging, owing to the intricacy of biological systems, inconsistencies in data analysis workflows, and gaps in available microbial reference databases. Moreover, the limited understanding of the metabolic interactions within LAB consortia in vegetable-based substrates hinders the ability to accurately predict shifts in bioactive compound profiles—a critical factor for developing functional food products.

Recent advances in analytical methods and data analysis may enhance predictions of the nutritional and sensory quality of fermented foods [140,141]. The integration of bioinformatics tools and machine learning algorithms will be essential in future studies, improving the interpretation of multi-omics data and enabling more accurate forecasts of microbial performance and fermentation outcomes. The industrial application of multi-omics will require standardized methodologies to optimize fermentation processes by considering interactions between raw materials, microbial consortia, and environmental factors. Ultimately, the integration of multi-omics, functional genomics, and transcriptomic data will be central to enhancing the nutritional, sensory, and functional characteristics of fermented vegetables, while also tackling issues of product uniformity and safety [140].

Additionally, advances in the development of recombinant microbial strains are poised to play a significant role in improving the efficiency of biotransformation processes in vegetable fermentation. Although conventional fermentation depends on the native metabolic potential of microorganisms, genetic engineering techniques have facilitated the creation of recombinant strains with enhanced productivity, stability, and substrate specificity. For example, genetically modified *Pseudomonas* strains have been employed to convert ferulic acid derived from cereal bran into bio-vanillin, though production is constrained by the subsequent oxidation of vanillin to vanillic acid. To overcome this challenge, recombinant *Escherichia coli* JM109, harboring vanillin-producing genes from *Pseudomonas fluorescens* BF13, has demonstrated increased bio-vanillin production by mitigating oxidation issues. Similarly, *P. fluorescens* BF13 strains with deletions in vanillin-oxidizing genes have further improved yields compared to their wild-type counterparts. Nevertheless, the industrial-scale application of recombinant fermentation faces multiple obstacles, including feedback inhibition, product instability, and the considerable expenses involved in downstream processing. Additionally, the cost and availability of developing recombinant strains pose barriers, particularly for small-scale enterprises. Future research should prioritize refining bioprocess optimization, enhancing microbial strain resilience, and incorporating multi-omics frameworks to unravel the regulatory mechanisms governing fermentation performance [142]. The convergence of synthetic biology, multi-omics, machine learning, and metabolic engineering holds considerable promise for advancing microbial fermentation in sustainable food systems; however, overcoming regulatory hurdles and economic constraints will be essential for its broader implementation.

## 8. Discussions and Conclusions

Microbial fermentation offers a green solution for managing food waste (FW), particularly for vegetable by-products, by reducing environmental impact, resource consumption, and greenhouse gas emissions compared to standard waste management methods such as landfilling. This approach can substantially reduce the demand for land, water, and energy, while lowering greenhouse gas emissions. Recent research shows that combining food waste fermentation with membrane separation results in a considerably lower global warming potential (GWP) of 164.1 kg CO_2_-eq per metric ton of food waste, making it a more eco-friendly alternative to landfilling. Therefore, fermentation emerges as an effective strategy for transforming organic residues into useful upgraded products, promoting environmental sustainability [143,144].

From an economic perspective, the fermentation of vegetable by-products entails costs associated with raw materials, equipment, labor, and downstream processing [145]. Although it requires substantial initial investment, fermentation adds economic value by reducing food losses and enhancing the marketability of raw materials [146]. Certifying the safety and quality of industrial fermentation processes further boosts consumer confidence and their willingness to pay higher prices for these products. However, downstream processing represents a major cost driver, often exceeding 60% of total expenses due to the complexity of separation and purification techniques. Integrating fermentation with continuous product removal can help mitigate costs and improve yield. The overall value addition in vegetable by-products fermentation depends on multiple factors, including the value of raw materials, processing expenses, logistics, and regulatory frameworks [146].

Utilizing by-products as a fermentation substrate significantly reduces raw material supply costs. However, ensuring the production of high-value products requires effective management of by-products to maintain the integrity of raw materials and prevent deterioration. Contamination risks increase when low-quality raw materials are used, particularly those with high initial levels of bacteria, fungi, or their toxins. Inadequate raw material quality compromises the safe consumption of the end product, particularly in processes conducted without heat treatment, such as fermentation [9].

The conversion of vegetable by-products into valuable products through fermentation is a strategy that could be implemented relatively quickly in industrialized nations. However, the same principle faces challenges in underdeveloped and developing countries due to several constraints. Scaling up fermentation from household to industrial levels in these areas is limited by the dependence of small producers on traditional practices and their reluctance to adopt process innovations. Furthermore, the lack of specialized knowledge and the high initial investment required for industrial fermentation pose substantial barriers to scaling up production [146]. In some areas, fermentation techniques are transmitted orally across generations, limiting the broader dissemination of knowledge and adherence to Good Manufacturing Practices (GMPs). As a result, fermented products are frequently marketed under poor hygienic conditions, with substandard packaging that heightens the risk of microbial spoilage and contamination [9].

Nevertheless, expanding small-scale fermentation remains essential to enhance the economic and social advantages of fermented foods and to facilitate sustainable growth of communities in low- and middle-income countries. Establishing producer associations could facilitate access to capital for acquiring equipment and other necessary resources while providing leverage to influence the regulatory environment [146]. A successful example of this approach can be found in Thailand, where the consortium model applied to soy sauce fermentation has demonstrated the potential of collaborative strategies [147]. To further support the scaling up of fermentation, governments should promote grassroots training programs and offer subsidies to establish small-scale enterprises focused on the development of safe and high-standard fermented products in rural areas [142].

Other examples of upscaling can be found in Africa, where the growing popularity of traditional African fermented foods has driven large commercial enterprises to standardize and upscale these processes through controlled production methods. However, the reliance on simplified microbial communities in industrial production often results in products that lack the rich flavors, functional properties, and health benefits of traditional counterparts. Despite these limitations, the increasing industrial interest highlights the strong consumer demand for fermented foods [146]. A notable example is Mahewu, a well-known maize- and sorghum-based fermented non-alcoholic beverage from Zimbabwe, available in both traditional homemade and commercial variants. Consumer interviews indicate a strong preference for the traditional version, attributed to its more complex aroma [9,146]. This suggests that, when upscaling fermentation processes, it is essential to consider the knowledge and preferences of both original producers and consumers to align product characteristics with consumer expectations.

Data on vegetable waste quantities in developing countries remain scarce, especially concerning the primary categories of waste produced in these areas. This gap is largely due to the dispersed nature of food processing in these regions and the absence of structured institutions overseeing food production, resulting in a lack of comprehensive data collection [9].

Consumer demand and acceptance are also key factors influencing the expansion of fermented vegetable production. The long-standing presence of fermentation practices in different food sectors has fostered widespread consumer acceptance of both microbial processes and fermented foods. Additionally, increasing consumer preference for natural alternatives over synthetic additives has further heightened appreciation for microbial fermentation, particularly due to its potential to replace chemical preservatives with natural bio-preservatives [148]. Moreover, the role of customs, culture, and religion in shaping traditionally fermented foods should not be overlooked. Indigenous communities have historically influenced fermentation practices, underscoring the importance of preserving traditional knowledge in the industrialization of fermented products [9].

Several studies have explored consumer acceptance of food developed from by-products, providing insights into their commercial potential. Perito et al. [149] examined the willingness to accept (WTA) foods containing olive by-products among 289 Italian consumers, identifying education, age, and sustainability attitudes as key influencing factors. Women possessing formal education and men aged between 40 and 60 exhibited higher acceptance, with age playing a significant role. While technophobia (the distrust about emerging technological approaches in food manufacturing) negatively affected WTA due to concerns about new food technologies, neophobia (the wariness of new food products) had little impact, likely due to Italian consumers’ familiarity with olive-based products. Acceptance was primarily influenced by the production process rather than the final product, particularly for familiar foods such as pasta, bread, and snacks. Consumers with strong sustainability awareness were more receptive, especially when these products were marketed with environmental and health benefits. These results highlight the commercial potential of by-product-derived foods, especially in product categories like baked goods, cheeses, and pasta [149]. Nitzko [150] investigated end-user acceptance of plant-origin food and animal by-products through an online survey of 614 German consumers. The study identified four consumer segments: low acceptance (5.9%), medium acceptance (34.3%), acceptance of plant by-products (15.3%), and high acceptance (44.5%). Consumers with low acceptance tend to be older, value sustainability, but require greater awareness of the benefits of by-product-based foods. Those accepting plant by-products are typically younger, often vegetarians or vegans, with high sustainability awareness. The largest group, with high acceptance, associates by-product-based foods with sustainability and health, demonstrating openness to both plant and animal by-products. The study suggests that targeted marketing strategies emphasizing sustainability and food safety can enhance acceptance, particularly among consumers with medium or low acceptance [150].

In conclusion, researchers have explored various fermentation methods and strains applied to vegetable processing by-products, resulting in the creation of novel fermented products with promising nutritional, health, and technological benefits. Each type of fermented vegetable offers unique characteristics and diverse applications, including food fortification and the creation of tailored products for consumers with specific dietary needs. Moreover, these studies demonstrate that bioprocessing vegetable by-products through fermentation represents a viable approach, combining by-product recycling with consumer demand for healthy foods. It serves as an effective and eco-friendly means to convert vegetable by-products into new ingredients, thereby boosting the presence of health-enhancing molecules. However, economic viability and scalability remain significant challenges in developing and less developed countries. Reducing processing costs, adopting scientifically optimized fermentation techniques, and improving product recovery strategies will be critical to widespread implementation. With continued research and supportive policies, the fermentation of vegetable by-products could play a substantial role in advancing global sustainability objectives. Moreover, a review of the literature reveals that most studies have focused on certain plant families, such as Poaceae and Solanaceae, due to the large quantities of by-products they produce annually and their well-known, easily exploitable potential. Accordingly, future research should prioritize the fermentation of a wider variety of plant families, starting with the most commonly consumed crops that generate large volumes of processing by-products.

Lastly, future vegetable fermentation research will be driven by the integration of multi-omics approaches, combining transcriptomics, proteomics, and metabolomics to gain deeper insights into microbial metabolism and fermentation dynamics. Moreover, progress in recombinant microbial technologies, especially in strains tailored for biotransformation, may offer valuable opportunities to enhance fermentation performance.

## Data Availability

The original contributions presented in this study are included in the article. Further inquiries can be directed to the corresponding author.

## Abbreviations

The following abbreviations are used in this manuscript:

FAO	Food and Agriculture Organization of the United Nations
USDA	United States Department of Agriculture
EPA	United States Environmental Protection Agency
BP	Billion pounds
AD	Anaerobic Digestion
MAE	Microwave-assisted extraction
SFE	Supercritical fluid extraction
UAE	Ultrasound-assisted extraction
HHP	High-pressure processing
EPS	Exopolysaccharides
SSF	Solid-state fermentation
SmF	Submerged fermentation
Imp	Mechanical index
LAB	Lactic acid bacteria
NSLAB	Non-starter lactic acid bacteria
AAB	Acetic acid bacteria
DPPH	2,2-Diphenyl-1-picrylhydrazyl
FRAP	Ferric Reducing Antioxidant Power
DPP-IV	Dipeptidyl peptidase-IV
T2D	Type 2 diabetes mellitus
ROS	Reactive oxygen species
GLS	Glucosinolates
ITC	Isothiocyanates
GAE	Gallic Acid Equivalent
QE	Quercetin Equivalents
CRC	Colorectal cancer
IL-8	Interleukin-8
ORAC	Oxygen Radical Absorbance Capacity
TPC	Total phenolic content
WHC	Water-holding capacity
SCFA	Short-chain fatty acids
FW	Food waste
GWP	Global warming potential
GMP	Good Manufacturing Practices
WTA	Willingness to accept

## References

[1] FAO Sustainable Development Goals Data Portal. https://www.fao.org/sustainable-development-goals-data-portal/data/.

[2] Sagar N.A., Pareek S., Sharma S., Yahia E.M., Lobo M.G. (2018). Fruit and Vegetable Waste: Bioactive Compounds, Their Extraction, and Possible Utilization. Compr. Rev. Food Sci. Food Saf..

[3] Zhu Y., Luan Y., Zhao Y., Liu J., Duan Z., Ruan R. (2023). Current Technologies and Uses for Fruit and Vegetable Wastes in a Sustainable System: A Review. Foods.

[4] FAO (2019). The State of Food and Agriculture 2019—Moving Forward on Food Loss and Waste Reduction.

[5] Kowalska H., Czajkowska K., Cichowska J., Lenart A. (2017). What’s New in Biopotential of Fruit and Vegetable by-Products Applied in the Food Processing Industry. Trends Food Sci. Technol..

[6] Wild F., Czerny M., Janssen A.M., Kole A.P.W., Marija Z., Domig K.J. (2014). The Evolution of a Plant-Based Alternative to Meat. Agro Food Ind. Hi Tech.

[7] FAO (2011). Global Food Losses and Food Waste—Extent, Causes and Prevention.

[8] Garrido-Galand S., Asensio-Grau A., Calvo-Lerma J., Heredia A., Andrés A. (2021). The Potential of Fermentation on Nutritional and Technological Improvement of Cereal and Legume Flours: A Review. Food Res. Int..

[9] Skowron K., Budzyńska A., Grudlewska-Buda K., Wiktorczyk-Kapischke N., Andrzejewska M., Wałecka-Zacharska E., Gospodarek-Komkowska E. (2022). Two Faces of Fermented Foods—The Benefits and Threats of Its Consumption. Front. Microbiol..

[10] Mannaa M., Han G., Seo Y.-S., Park I. (2021). Evolution of Food Fermentation Processes and the Use of Multi-Omics in Deciphering the Roles of the Microbiota. Foods.

[11] Marsh A.J., Hill C., Ross R.P., Cotter P.D. (2014). Fermented Beverages with Health-Promoting Potential: Past and Future Perspectives. Trends Food Sci. Technol..

[12] Liu L., Wang J., Rosenberg D., Zhao H., Lengyel G., Nadel D. (2018). Fermented Beverage and Food Storage in 13,000 y-old Stone Mortars at Raqefet Cave, Israel: Investigating Natufian Ritual Feasting. J. Archaeol. Sci. Rep..

[13] Đorđević T.M., Šiler-Marinković S.S., Dimitrijević-Branković S.I. (2010). Effect of Fermentation on Antioxidant Properties of Some Cereals and Pseudo Cereals. Food Chem..

[14] Thirunathan P., Manickavasagan A. (2019). Processing Methods for Reducing Alpha-Galactosides in Pulses. Crit. Rev. Food Sci. Nutr..

[15] Adebo O.A., Njobeh P.B., Adebiyi J.A., Gbashi S., Phoku J.Z., Kayitesi E. (2017). Fermented Pulse-Based Food Products in Developing Nations as Functional Foods and Ingredients. Functional Food—Improve Health Through Adequate Food.

[16] Saharan P., Sadh P.K., Duhan J.S. (2017). Comparative Assessment of Effect of Fermentation on Phenolics, Flavanoids and Free Radical Scavenging Activity of Commonly Used Cereals. Biocatal. Agric. Biotechnol..

[17] Xiang H., Sun-Waterhouse D., Waterhouse G.I.N., Cui C., Ruan Z. (2019). Fermentation-Enabled Wellness Foods: A Fresh Perspective. Food Sci. Hum. Wellness.

[18] Onweluzo J.C., Nwabugwu C.C. (2009). Fermentation of Millet (*Pennisetum americanum*) and Pigeon Pea (*Cajanus cajan*) Seeds for Flour Production: Effects on Composition and Selected Functional Properties. Pak. J. Nutr..

[19] Onimawo I.A., Nmerole E.C., Idoko P.I., Akubor P.I. (2003). Effects of Fermentation on Nutrient Content and Some Functional Properties of Pumpkin Seed (*Telfaria occidentalis*). Plant Foods Hum. Nutr..

[20] Olagunju A.I., Ifesan B.O.T. (2013). Changes in Nutrient and Antinutritional Contents of Sesame Seeds during Fermentation. J. Microbiol. Biotechnol. Food Sci..

[21] Xing Q., Dekker S., Kyriakopoulou K., Boom R.M., Smid E.J., Schutyser M.A.I. (2020). Enhanced Nutritional Value of Chickpea Protein Concentrate by Dry Separation and Solid State Fermentation. Innov. Food Sci. Emerg. Technol..

[22] Marco M.L., Heeney D., Binda S., Cifelli C.J., Cotter P.D., Foligné B., Gänzle M., Kort R., Pasin G., Pihlanto A. (2017). Health Benefits of Fermented Foods: Microbiota and Beyond. Curr. Opin. Biotechnol..

[23] De Roos J., De Vuyst L. (2018). Acetic Acid Bacteria in Fermented Foods and Beverages. Curr. Opin. Biotechnol..

[24] Anal A.K. (2019). Quality Ingredients and Safety Concerns for Traditional Fermented Foods and Beverages from Asia: A Review. Fermentation.

[25] Filannino P., Di Cagno R., Gobbetti M. (2018). Metabolic and Functional Paths of Lactic Acid Bacteria in Plant Foods: Get out of the Labyrinth. Curr. Opin. Biotechnol..

[26] Tlais A.Z.A., Fiorino G.M., Polo A., Filannino P., Cagno R. (2020). Di High-Value Compounds in Fruit, Vegetable and Cereal Byproducts: An Overview of Potential Sustainable Reuse and Exploitation. Molecules.

[27] Dey T.B., Chakraborty S., Jain K.K., Sharma A., Kuhad R.C. (2016). Antioxidant Phenolics and Their Microbial Production by Submerged and Solid State Fermentation Process: A Review. Trends Food Sci. Technol..

[28] Ricci A., Bernini V., Maoloni A., Cirlini M., Galaverna G., Neviani E., Lazzi C. (2019). Vegetable By-Product Lacto-Fermentation as a New Source of Antimicrobial Compounds. Microorganisms.

[29] Kagkli D.M., Corich V., Bovo B., Lante A., Giacomini A. (2016). Antiradical and Antimicrobial Properties of Fermented Red Chicory (*Cichorium intybus* L.) by-Products. Ann. Microbiol..

[30] Pandey A. (2003). Solid-State Fermentation. Biochem. Eng. J..

[31] Sadh P.K., Duhan S., Duhan J.S. (2018). Agro-Industrial Wastes and Their Utilization Using Solid State Fermentation: A Review. Bioresour. Bioprocess..

[32] Krishna C. (2008). Solid-State Fermentation Systems—An Overview. Crit. Rev. Biotechnol..

[33] Srivastava N., Srivastava M., Ramteke P.W., Mishra P.K. (2019). Solid-State Fermentation Strategy for Microbial Metabolites Production: An Overview. New and Future Developments in Microbial Biotechnology and Bioengineering.

[34] Vassileva M., Malusà E., Sas-Paszt L., Trzcinski P., Galvez A., Flor-Peregrin E., Shilev S., Canfora L., Mocali S., Vassilev N. (2021). Fermentation Strategies to Improve Soil Bio-Inoculant Production and Quality. Microorganisms.

[35] Singhania R.R., Patel A.K., Soccol C.R., Pandey A. (2009). Recent Advances in Solid-State Fermentation. Biochem. Eng. J..

[36] Mitchell D.A., Berovič M., Krieger N., Mitchell D.A., Berovič M., Krieger N. (2006). Solid-State Fermentation Bioreactors.

[37] Liu X., Kokare C. (2017). Microbial Enzymes of Use in Industry. Biotechnology of Microbial Enzymes.

[38] Soccol C.R., da Costa E.S.F., Letti L.A.J., Karp S.G., Woiciechowski A.L., de Souza Vandenberghe L.P. (2017). Recent Developments and Innovations in Solid State Fermentation. Biotechnol. Res. Innov..

[39] Robinson T., Singh D., Nigam P. (2001). Solid-State Fermentation: A Promising Microbial Technology for Secondary Metabolite Production. Appl. Microbiol. Biotechnol..

[40] Zhang Y., Wang L., Chen H. (2017). Correlations of Medium Physical Properties and Process Performance in Solid-State Fermentation. Chem. Eng. Sci..

[41] Ravindran R., Hassan S.S., Williams G.A., Jaiswal A.K. (2018). A Review on Bioconversion of Agro-Industrial Wastes to Industrially Important Enzymes. Bioengineering.

[42] Satora P., Skotniczny M., Strnad S., Piechowicz W. (2021). Chemical Composition and Sensory Quality of Sauerkraut Produced from Different Cabbage Varieties. LWT.

[43] Yang X., Hu W., Xiu Z., Jiang A., Yang X., Sarengaowa, Ji Y., Guan Y., Feng K. (2020). Microbial Dynamics and Volatilome Profiles during the Fermentation of Chinese Northeast Sauerkraut by *Leuconostoc mesenteroides* ORC 2 and *Lactobacillus plantarum* HBUAS 51041 under Different Salt Concentrations. Food Res. Int..

[44] Yang Y., Fan Y., Li T., Yang Y., Zeng F., Wang H., Suo H., Song J., Zhang Y. (2022). Microbial Composition and Correlation between Microbiota and Quality-Related Physiochemical Characteristics in Chongqing Radish Paocai. Food Chem..

[45] Tan X., Cui F., Wang D., Lv X., Li X., Li J. (2023). Fermented Vegetables: Health Benefits, Defects, and Current Technological Solutions. Foods.

[46] Zhang C., Zhang J., Liu D. (2021). Biochemical Changes and Microbial Community Dynamics during Spontaneous Fermentation of Zhacai, a Traditional Pickled Mustard Tuber from China. Int. J. Food Microbiol..

[47] Maoloni A., Ferrocino I., Milanović V., Cocolin L., Corvaglia M.R., Ottaviani D., Bartolini C., Talevi G., Belleggia L., Cardinali F. (2020). The Microbial Diversity of Non-Korean Kimchi as Revealed by Viable Counting and Metataxonomic Sequencing. Foods.

[48] Xiao Z.B., Zhu J.C., Feng T., Tian H.X., Yu H.Y., Niu Y.W., Zhang X.M. (2010). Comparison of Volatile Components in Chinese Traditional Pickled Peppers Using HS–SPME–GC–MS, GC–O and Multivariate Analysis. Nat. Prod. Res..

[49] Yuan L., Xu F., Xu Y., Wu J., Lao F. (2022). Production of Marinated Chinese Lotus Root Slices Using High-Pressure Processing as an Alternative to Traditional Thermal-and-Soaking Procedure. Molecules.

[50] Wang Z.-P., Wang Q.-Q., Liu S., Liu X.-F., Yu X.-J., Jiang Y.-L. (2019). Efficient Conversion of Cane Molasses Towards High-Purity Isomaltulose and Cellular Lipid Using an Engineered *Yarrowia lipolytica* Strain in Fed-Batch Fermentation. Molecules.

[51] Chen Y., Chen L., Liu L., Bi X., Liu X. (2023). Characteristics of Microbial Communities in Fermentation of Pickled Ginger and Their Correlation with Its Volatile Flavors. Food Biosci..

[52] Moore J.F., DuVivier R., Johanningsmeier S.D. (2022). Changes in the Free Amino Acid Profile of Pickling Cucumber during Lactic Acid Fermentation. J. Food Sci..

[53] Janiszewska-Turak E., Tracz K., Bielińska P., Rybak K., Pobiega K., Gniewosz M., Woźniak Ł., Gramza-Michałowska A. (2022). The Impact of the Fermentation Method on the Pigment Content in Pickled Beetroot and Red Bell Pepper Juices and Freeze-Dried Powders. Appl. Sci..

[54] Cardinali F., Botta C., Harasym J., Ferrocino I., Reale A., Boscaino F., Di Renzo T., Milanović V., Garofalo C., Rampanti G. (2024). Lacto-Fermented Garlic Handcrafted in the Lower Silesia Region (Poland): Microbial Diversity, Morpho-Textural Traits, and Volatile Compounds. Food Res. Int..

[55] Montaño A., Casado F.J., de Castro A., Sánchez A.H., Rejano L. (2004). Vitamin Content and Amino Acid Composition of Pickled Garlic Processed with and without Fermentation. J. Agric. Food Chem..

[56] Fayek N.M., Farag M.A., Saber F.R. (2021). Metabolome Classification via GC/MS and UHPLC/MS of Olive Fruit Varieties Grown in Egypt Reveal Pickling Process Impact on Their Composition. Food Chem..

[57] Shang Z., Li M., Zhang W., Cai S., Hu X., Yi J. (2022). Analysis of Phenolic Compounds in Pickled Chayote and Their Effects on Antioxidant Activities and Cell Protection. Food Res. Int..

[58] Maoloni A., Milanović V., Osimani A., Cardinali F., Garofalo C., Belleggia L., Foligni R., Mannozzi C., Mozzon M., Cirlini M. (2021). Exploitation of Sea Fennel (*Crithmum maritimum* L.) for Manufacturing of Novel High-Value Fermented Preserves. Food Bioprod. Process..

[59] Estrella M.J., Pieckenstain F.L., Marina M., Diaz L.E., Ruiz O.A. (2004). Cheese Whey: An Alternative Growth and Protective Medium for *Rhizobium loti* Cells. J. Ind. Microbiol. Biotechnol..

[60] Singh A.K., Bhatt R.P., Pant S. (2011). Optimization and Comparative Study of the Sugar Waste for the Growth of Rhizobium Cells Along with Traditional Laboratory Media. Res. J. Microbiol..

[61] Suryanarayan S. (2003). Current Industrial Practice in Solid State Fermentations for Secondary Metabolite Production: The Biocon India Experience. Biochem. Eng. J..

[62] Ouedraogo J.-P., Tsang A. (2021). Production of Native and Recombinant Enzymes by Fungi for Industrial Applications. Encyclopedia of Mycology.

[63] Mehra S., Chandrawanshi V., Prashad K. (2017). Advanced Bioprocess Engineering: Fed-Batch and Perfusion Processes. Applied Bioengineering.

[64] Kapoor M., Panwar D., Kaira G.S. (2016). Bioprocesses for Enzyme Production Using Agro-Industrial Wastes. Agro-Industrial Wastes as Feedstock for Enzyme Production.

[65] Lim H.C., Shin H.S. (2013). Fed-Batch Cultures: Principles and Applications of Semi-Batch Bioreactors.

[66] Damir O., Mladen P., Božidar S., Srñan N. (2011). Cultivation of the Bacterium Azotobacter Chroococcum for Preparation of Biofertilizers. Afr. J. Biotechnol..

[67] Siddiqui S.A., Erol Z., Rugji J., Taşçı F., Kahraman H.A., Toppi V., Musa L., Di Giacinto G., Bahmid N.A., Mehdizadeh M. (2023). An Overview of Fermentation in the Food Industry—Looking Back from a New Perspective. Bioresour. Bioprocess..

[68] Aleman R., Montero-Fernández I., Marcía J., Maldonado S.S., Martín-Vertedor D. (2024). Application of Fermentation as a Strategy for the Transformation and Valorization of Vegetable Matrices. Fermentation.

[69] Khalisanni K. (2011). An Overview of Lactic Acid Bacteria. Int. J. Biosci..

[70] Quinto E.J., Jiménez P., Caro I., Tejero J., Mateo J., Girbés T. (2014). Probiotic Lactic Acid Bacteria: A Review. Food Nutr. Sci..

[71] Ivanov V. (2020). Environmental Microbiology for Engineers.

[72] Axelsson L. (2004). Lactic Acid Bacteria: Classification and Physiology. Food Sci. Technol..

[73] Saadoun J.H., Bertani G., Levante A., Vezzosi F., Ricci A., Bernini V., Lazzi C. (2021). Fermentation of Agri-Food Waste: A Promising Route for the Production of Aroma Compounds. Foods.

[74] Coelho M.C., Malcata F.X., Silva C.C.G. (2022). Lactic Acid Bacteria in Raw-Milk Cheeses: From Starter Cultures to Probiotic Functions. Foods.

[75] Leroy F., De Vuyst L. (2004). Lactic Acid Bacteria as Functional Starter Cultures for the Food Fermentation Industry. Trends Food Sci. Technol..

[76] Zheng J., Wittouck S., Salvetti E., Franz C.M.A.P., Harris H.M.B., Mattarelli P., O’Toole P.W., Pot B., Vandamme P., Walter J. (2020). A Taxonomic Note on the Genus *Lactobacillus*: Description of 23 Novel Genera, Emended Description of the Genus *Lactobacillus* Beijerinck 1901, and Union of *Lactobacillaceae* and *Leuconostocaceae*. Int. J. Syst. Evol. Microbiol..

[77] Ramires F.A., Bavaro A.R., D’Antuono I., Linsalata V., D’Amico L., Baruzzi F., Pinto L., Tarantini A., Garbetta A., Cardinali A. (2023). Liquid Submerged Fermentation by Selected Microbial Strains for Onion Skins Valorization and Its Effects on Polyphenols. World J. Microbiol. Biotechnol..

[78] Khubber S., Marti-Quijal F.J., Tomasevic I., Remize F., Barba F.J. (2022). Lactic Acid Fermentation as a Useful Strategy to Recover Antimicrobial and Antioxidant Compounds from Food and By-Products. Curr. Opin. Food Sci..

[79] Kimoto-Nira H., Ohashi Y., Amamiya M., Moriya N., Ohmori H., Sekiyama Y. (2020). Fermentation of Onion (*Allium cepa* L.) Peel by Lactic Acid Bacteria for Production of Functional Food. J. Food Meas. Charact..

[80] Tsangalis D., Ashton J.F., Mcgill A.E.J., Shah N.P. (2002). Enzymic Transformation of Isoflavone Phytoestrogens in Soymilk by Β-Glucosidase-Producing Bifidobacteria. J. Food Sci..

[81] Qiu X., Zhang Y., Hong H. (2021). Classification of Acetic Acid Bacteria and Their Acid Resistant Mechanism. AMB Express.

[82] Merli G., Becci A., Amato A., Beolchini F. (2021). Acetic Acid Bioproduction: The Technological Innovation Change. Sci. Total Environ..

[83] Sabater C., Ruiz L., Delgado S., Ruas-Madiedo P., Margolles A. (2020). Valorization of Vegetable Food Waste and By-Products Through Fermentation Processes. Front. Microbiol..

[84] Karel K. (2006). The Family Acetobacteraceae: The Genera *Acetobacter*, *Acidomonas*, *Asaia*, *Gluconacetobacter*, *Gluconobacter*, and *Kozakia*. Prokaryotes.

[85] Lynch K.M., Zannini E., Wilkinson S., Daenen L., Arendt E.K. (2019). Physiology of Acetic Acid Bacteria and Their Role in Vinegar and Fermented Beverages. Compr. Rev. Food Sci. Food Saf..

[86] Misra H.S., Rajpurohit Y.S., Khairnar N.P. (2012). Pyrroloquinoline-Quinone and Its Versatile Roles in Biological Processes. J. Biosci..

[87] Sainz F., Navarro D., Mateo E., Torija M.J., Mas A. (2016). Comparison of D-Gluconic Acid Production in Selected Strains of Acetic Acid Bacteria. Int. J. Food Microbiol..

[88] Raspor P., Goranovič D. (2008). Biotechnological Applications of Acetic Acid Bacteria. Crit. Rev. Biotechnol..

[89] Sengun I.Y. (2017). Acetic Acid Bacteria: Fundamentals and Food Applications.

[90] Li Y., He D., Niu D., Zhao Y. (2015). Acetic Acid Production from Food Wastes Using Yeast and Acetic Acid Bacteria Micro-Aerobic Fermentation. Bioprocess. Biosyst. Eng..

[91] Gomes R.J., de Fatima Borges M., de Freitas Rosa M., Castro-Gómez R.J.H., Spinosa W.A. (2018). Acetic Acid Bacteria in the Food Industry: Systematics, Characteristics and Applications. Food Technol. Biotechnol..

[92] Vegas C., Mateo E., González Á., Jara C., Guillamón J.M., Poblet M., Torija M.J., Mas A. (2010). Population Dynamics of Acetic Acid Bacteria during Traditional Wine Vinegar Production. Int. J. Food Microbiol..

[93] Gullo M., Verzelloni E., Canonico M. (2014). Aerobic Submerged Fermentation by Acetic Acid Bacteria for Vinegar Production: Process and Biotechnological Aspects. Process Biochem..

[94] Alexandri M., Neu A., Schneider R., López-Gómez J.P., Venus J. (2019). Evaluation of Various *Bacillus coagulans* Isolates for the Production of High Purity L-lactic Acid Using Defatted Rice Bran Hydrolysates. Int. J. Food Sci. Technol..

[95] Cole M.R., Eggleston G., Gaines D.K., Heckemeyer M. (2019). Development of an Enzyme Cocktail to Bioconvert Untapped Starch in Sweet Sorghum Processing By-Products: Part I. Ind. Crops Prod..

[96] Mukherjee R., Chakraborty R., Dutta A. (2019). Comparison of Optimization Approaches (Response Surface Methodology and Artificial Neural Network-genetic Algorithm) for a Novel Mixed Culture Approach in Soybean Meal Fermentation. J. Food Process Eng..

[97] Tomita H., Okazaki F., Tamaru Y. (2019). Biomethane Production from Sugar Beet Pulp under Cocultivation with Clostridium Cellulovorans and Methanogens. AMB Express.

[98] Maicas S. (2020). The Role of Yeasts in Fermentation Processes. Microorganisms.

[99] Sarris D., Rapti A., Papafotis N., Koutinas A.A., Papanikolaou S. (2019). Production of Added-Value Chemical Compounds through Bioconversions of Olive-Mill Wastewaters Blended with Crude Glycerol by a *Yarrowia lipolytica* Strain. Molecules.

[100] Gong S., Xie F., Lan X., Zhang W., Gu X., Wang Z. (2020). Effects of Fermentation on Compositions, Color, and Functional Properties of Gelatinized Potato Flours. J. Food Sci..

[101] Puligundla P., Smogrovicova D., Obulam V.S.R., Ko S. (2011). Very High Gravity (VHG) Ethanolic Brewing and Fermentation: A Research Update. J. Ind. Microbiol. Biotechnol..

[102] Müjdeci G.N., Khosravi-Darani K. (2022). Valorization of Fruit and Vegetable Waste: Yeast Fermentation. Fruits and Vegetable Wastes.

[103] Cebrián M., Ibarruri J. (2023). Filamentous Fungi Processing by Solid-State Fermentation. Current Developments in Biotechnology and Bioengineering.

[104] Beg Q.K., Kapoor M., Mahajan L., Hoondal G.S. (2001). Microbial Xylanases and Their Industrial Applications: A Review. Appl. Microbiol. Biotechnol..

[105] Neira-Vielma A.A., Aguilar C.N., Ilyina A., Contreras-Esquivel J.C., das Graça Carneiro-da-Cunha M., Michelena-Álvarez G., Martínez-Hernández J.L. (2018). Purification and Biochemical Characterization of an Aspergillus Niger Phytase Produced by Solid-State Fermentation Using Triticale Residues as Substrate. Biotechnol. Rep..

[106] Mathew J.J., NK P.J.S., Vazhacharickal A.A., Sajeshkumar N.K., Ashokan A. (2016). Amylase Production by *Aspergillus niger* through Submerged Fermentation Using Starchy Food Byproducts as Substrate. Int. J. Herb. Med..

[107] Melnichuk N., Braia M.J., Anselmi P.A., Meini M.-R., Romanini D. (2020). Valorization of Two Agroindustrial Wastes to Produce Alpha-Amylase Enzyme from *Aspergillus oryzae* by Solid-State Fermentation. Waste Manag..

[108] Novelli P.K., Barros M.M., Fleuri L.F. (2016). Novel Inexpensive Fungi Proteases: Production by Solid State Fermentation and Characterization. Food Chem..

[109] Oliveira F., Moreira C., Salgado J.M., Abrunhosa L., Venâncio A., Belo I. (2016). Olive Pomace Valorization by *Aspergillus* Species: Lipase Production Using Solid-state Fermentation. J. Sci. Food Agric..

[110] Amorim C., Silvério S.C., Rodrigues L.R. (2019). One-Step Process for Producing Prebiotic Arabino-Xylooligosaccharides from Brewer’s Spent Grain Employing Trichoderma Species. Food Chem..

[111] Costa J.R., Tonon R.V., Gottschalk L.M., Santiago M.C.D.A., Mellinger-Silva C., Pastrana L., Pintado M.M., Cabral L.M. (2019). Enzymatic Production of Xylooligosaccharides from Brazilian Syrah Grape Pomace Flour: A Green Alternative to Conventional Methods for Adding Value to Agricultural By-Products. J. Sci. Food Agric..

[112] Paz A., Outeiriño D., Guerra N.P., Domínguez J.M. (2019). Enzymatic Hydrolysis of Brewer’s Spent Grain to Obtain Fermentable Sugars. Bioresour. Technol..

[113] Ganaie M.A., Soni H., Naikoo G.A., Oliveira L.T.S., Rawat H.K., Mehta P.K., Narain N. (2017). Screening of Low Cost Agricultural Wastes to Maximize the Fructosyltransferase Production and Its Applicability in Generation of Fructooligosaccharides by Solid State Fermentation. Int. Biodeterior. Biodegrad..

[114] Muñiz-Márquez D.B., Teixeira J.A., Mussatto S.I., Contreras-Esquivel J.C., Rodríguez-Herrera R., Aguilar C.N. (2019). Fructo-Oligosaccharides (FOS) Production by Fungal Submerged Culture Using Aguamiel as a Low-Cost by-Product. LWT.

[115] Strong P.J., Self R., Allikian K., Szewczyk E., Speight R., O’Hara I., Harrison M.D. (2022). Filamentous Fungi for Future Functional Food and Feed. Curr. Opin. Biotechnol..

[116] Gmoser R., Sintca C., Taherzadeh M.J., Lennartsson P.R. (2019). Combining Submerged and Solid State Fermentation to Convert Waste Bread into Protein and Pigment Using the Edible Filamentous Fungus *N. intermedia*. Waste Manag..

[117] Intasit R., Cheirsilp B., Suyotha W., Boonsawang P. (2021). Synergistic Production of Highly Active Enzymatic Cocktails from Lignocellulosic Palm Wastes by Sequential Solid State-Submerged Fermentation and Co-Cultivation of Different Filamentous Fungi. Biochem. Eng. J..

[118] Ricci A., Bertani G., Maoloni A., Bernini V., Levante A., Neviani E., Lazzi C. (2021). Antimicrobial Activity of Fermented Vegetable Byproduct Extracts for Food Applications. Foods.

[119] Ricci A., Marrella M., Saadoun J.H., Bernini V., Godani F., Dameno F., Neviani E., Lazzi C. (2020). Development of Lactic Acid-Fermented Tomato Products. Microorganisms.

[120] Serna-Barrera M.A., Bas-Bellver C., Seguí L., Betoret N., Barrera C. (2024). Exploring Fermentation with Lactic Acid Bacteria as a Pretreatment for Enhancing Antioxidant Potential in Broccoli Stem Powders. AIMS Microbiol..

[121] Lücke F.-K., Fritz V., Tannhäuser K., Arya A. (2019). Controlled Fermentation of Rapeseed Presscake by *Rhizopus*, and Its Effect on Some Components with Relevance to Human Nutrition. Food Res. Int..

[122] Pontonio E., Dingeo C., Gobbetti M., Rizzello C.G. (2019). Maize Milling By-Products: From Food Wastes to Functional Ingredients Through Lactic Acid Bacteria Fermentation. Front. Microbiol..

[123] Reque P.M., Pinilla C.M.B., Tinello F., Corich V., Lante A., Giacomini A., Brandelli A. (2020). Biochemical and Functional Properties of Wheat Middlings Bioprocessed by Lactic Acid Bacteria. J. Food Biochem..

[124] Rossi S., Gottardi D., Siroli L., Giordani B., Vitali B., Vannini L., Patrignani F., Lanciotti R. (2024). Functional and Biochemical Characterization of Pre-Fermented Ingredients Obtained by the Fermentation of Durum Wheat by-Products. J. Funct. Foods.

[125] Siroli L., Giordani B., Rossi S., Gottardi D., McMahon H., Augustyniak A., Menon A., Vannini L., Vitali B., Patrignani F. (2022). Antioxidant and Functional Features of Pre-Fermented Ingredients Obtained by the Fermentation of Milling By-Products. Fermentation.

[126] Pontonio E., Dingeo C., Di Cagno R., Blandino M., Gobbetti M., Rizzello C.G. (2020). Brans from Hull-Less Barley, Emmer and Pigmented Wheat Varieties: From by-Products to Bread Nutritional Improvers Using Selected Lactic Acid Bacteria and Xylanase. Int. J. Food Microbiol..

[127] Ritthibut N., Oh S.-J., Lim S.-T. (2021). Enhancement of Bioactivity of Rice Bran by Solid-State Fermentation with Aspergillus Strains. LWT.

[128] Moon S.-H., Chang H.-C. (2021). Rice Bran Fermentation Using Lactiplantibacillus Plantarum EM as a Starter and the Potential of the Fermented Rice Bran as a Functional Food. Foods.

[129] Yi C., Xie L., Cao Z., Quan K., Zhu H., Yuan J. (2022). Effects of Rice Bran Fermented with *Lactobacillus plantarum* on Palatability, Volatile Profiles, and Antioxidant Activity of Brown Rice Noodles. Int. J. Food Sci. Technol..

[130] Gong S., Yu Y., Li W., Wu J., Wang Z. (2022). Effects of Amylolytic Lactobacillus Fermentation on the Nutritional Quality and Digestibility of Purple Potato Flour. J. Food Compos. Anal..

[131] Efenberger-Szmechtyk M., Nowak A., Czyzowska A. (2021). Plant Extracts Rich in Polyphenols: Antibacterial Agents and Natural Preservatives for Meat and Meat Products. Crit. Rev. Food Sci. Nutr..

[132] Iga-Buitrón D., Torres-Maravilla E., Bermúdez-Humaran L.G., Ascacio-Valdes J.A., Rodríguez-Herrera R., Aguilar C.N., Flores-Gallegos A.C. (2023). Lactic Fermentation of Broccoli (*Brassica oleracea* Var. *italica*) to Enhance the Antioxidant and Antiproliferative Activities. Fermentation.

[133] FAO Cereal Supply and Demand Brief (2024). World Food Situation. Food and Agriculture Organization of the United Nations.

[134] Zamaratskaia G., Gerhardt K., Wendin K. (2021). Biochemical Characteristics and Potential Applications of Ancient Cereals—An Underexploited Opportunity for Sustainable Production and Consumption. Trends Food Sci. Technol..

[135] Gobbetti M., De Angelis M., Di Cagno R., Calasso M., Archetti G., Rizzello C.G. (2019). Novel Insights on the Functional/Nutritional Features of the Sourdough Fermentation. Int. J. Food Microbiol..

[136] Katina K., Juvonen R., Laitila A., Flander L., Nordlund E., Kariluoto S., Piironen V., Poutanen K. (2012). Fermented Wheat Bran as a Functional Ingredient in Baking. Cereal Chem..

[137] Noort M.W.J., van Haaster D., Hemery Y., Schols H.A., Hamer R.J. (2010). The Effect of Particle Size of Wheat Bran Fractions on Bread Quality—Evidence for Fibre–Protein Interactions. J. Cereal Sci..

[138] Zhang J., Liu M., Zhao Y., Zhu Y., Bai J., Fan S., Zhu L., Song C., Xiao X. (2022). Recent Developments in Fermented Cereals on Nutritional Constituents and Potential Health Benefits. Foods.

[139] Verni M., De Mastro G., De Cillis F., Gobbetti M., Rizzello C.G. (2019). Lactic Acid Bacteria Fermentation to Exploit the Nutritional Potential of Mediterranean Faba Bean Local Biotypes. Food Res. Int..

[140] Li Y., He W., Liu S., Hu X., He Y., Song X., Yin J., Nie S., Xie M. (2024). Innovative Omics Strategies in Fermented Fruits and Vegetables: Unveiling Nutritional Profiles, Microbial Diversity, and Future Prospects. Compr. Rev. Food Sci. Food Saf..

[141] Garcia C., Guerin M., Souidi K., Remize F. (2020). Lactic Fermented Fruit or Vegetable Juices: Past, Present and Future. Beverages.

[142] Rastogi Y.R., Thakur R., Thakur P., Mittal A., Chakrabarti S., Siwal S.S., Thakur V.K., Saini R.V., Saini A.K. (2022). Food Fermentation—Significance to Public Health and Sustainability Challenges of Modern Diet and Food Systems. Int. J. Food Microbiol..

[143] Muhammad N.I.S., Rosentrater K.A. (2020). Comparison of Global-Warming Potential Impact of Food Waste Fermentation to Landfill Disposal. SN Appl. Sci..

[144] Ayodele A., Chidinma D.U. (2024). Microbial Fermentation for Sustainable Food Production: Engineered Microorganisms for Alternative Proteins and Food Ingredients. Quantum J. Eng. Sci. Technol..

[145] Dhagat S., Jujjavarapu S.E. (2024). Economics of Fermentation Processes. Recent Advances in Bioprocess Engineering and Bioreactor Design.

[146] Materia V.C., Linnemann A.R., Smid E.J., Schoustra S.E. (2021). Upscaling of Traditional Fermented Foods to Build Value Chains and to Promote Women.

[147] Valyasevi R., Rolle R.S. (2002). An Overview of Small-Scale Food Fermentation Technologies in Developing Countries with Special Reference to Thailand: Scope for Their Improvement. Int. J. Food Microbiol..

[148] Capozzi V., Fragasso M., Bimbo F. (2021). Microbial Resources, Fermentation and Reduction of Negative Externalities in Food Systems: Patterns toward Sustainability and Resilience. Fermentation.

[149] Perito M.A., Di Fonzo A., Sansone M., Russo C. (2019). Consumer Acceptance of Food Obtained from Olive By-Products. Br. Food J..

[150] Nitzko S. (2023). Consumer Acceptance of the Use of Plant and Animal By-Products of Food Manufacturing for Human Nutrition. Food Humanit..

